# Sepsis-induced myocardial dysfunction diagnosed with strain versus non-strain echocardiography parameters: incidence, evolution and association with prognosis

**DOI:** 10.1186/s13613-025-01561-w

**Published:** 2025-09-25

**Authors:** Filipe André Gonzalez, Jacobo Bacariza, Ana Rita Varudo, João Leote, Ricardo Meireles Mateus, Cristina Maia Martins, Maria Inês Ribeiro, Filippo Sanfilippo, Luís Rocha Lopes, Ana G. Almeida

**Affiliations:** 1 Faculdade de Medicina, Centro Cardiovascular da Universidade de Lisboa, CCUL@RISE, Universidade de Lisboa, Av. Prof. Egas Moniz MB, Lisboa, 1649-028 Portugal; 2 Intensive Care Medicine at Intensive Care Department of Hospital Garcia de Orta, Av. Torrado da Silva, Almada, 2805-267 Portugal; 3 Intensive Care Unit of Hospital CUF Tejo, Lisboa, Portugal; 4 Department of Anesthesia and Intensive Care, “Policlinico-San Marco” University Hospital, Viale Carlo Azeglio Ciampi, Catania, 95121 CT Italy; 5 Department of Surgery and Medical-Surgical Specialties, Section of Anesthesia and Intensive Care, University of Catania, Piazza Università, 2, Catania, 95124 Italy; 6 Inherited Cardiac Disease Unit, Bart’s Heart Centre St Bartholomew’s Hospital, W Smithfield, London, EC1A 7BE UK; 7 Institute of Cardiovascular Science, University College London, 62 Huntley St, London, WC1E 6DD UK; 8 Cardiology Clinics, Heart and Vessels Department, ULS Santa Maria, Lisbon, Portugal

**Keywords:** Septic cardiomyopathy, Speckle tracking echocardiography (STE), Septic shock, Left ventricle, Right ventricle

## Abstract

**Background:**

Sepsis-induced myocardial dysfunction (SIMD) remains poorly defined due to scarce longitudinal studies with advanced echocardiography. We characterized SIMD progression using speckle tracking echocardiography (STE).

**Methods:**

Prospective single-center study in septic shock patients admitted to intensive care. SIMD was defined as any left ventricular (LV, systolic and/or diastolic) and/or right ventricular (RV) systolic dysfunction, using STE or non-STE criteria, on days 1, 7 and 30. We studied prevalence, evolution and prognosis of SIMD classified with either criteria using Cox regression.

**Results:**

Ninety-eight consecutive patients were included. On day 1, SIMD was identified in *n* = 57/98 (58.2%) and *n* = 70/98 (71.4%;*p* = 0.072) by non-STE and STE parameters, respectively. No significant difference in diagnosis was seen for LV diastolic dysfunction: *n* = 50/98 (51.0%, non-STE) vs. *n* = 51/98 (52.0%, STE; *p* = 1.00). Prevalences of LV and RV systolic dysfunction were not significantly higher with STE criteria: *n* = 59/98 (60.2%, STE) vs. *n* = 47/98 (48.0%, non-STE; *p* = 0.115) for LV; *n* = 39/98 (39.8%, STE) vs. *n* = 27/98 (27.6%, non-STE; *p* = 0.096) for RV. More patients recovered from SIMD when evaluated with non-STE criteria at day 7 (35.3% vs. 17.5% STE; *p* = 0.033), but not at day 30 (24.5% vs. 18.8% STE; *p* = 0.501). The 30-day mortality (*n* = 33/98, 33.7%) was associated with SIMD diagnosed using non-STE (*p* = 0.010), but not with STE (*p* = 0.057). In Cox regression, only LVDD by non-STE criteria predicted 30-day mortality (*p* = 0.005).

**Conclusions:**

The incidence of SIMD in septic shock is higher when using STE criteria, with lower reversibility in the first week. A broad definition of SIMD utilizing STE criteria does not seem to provide additional prognostic value.

***Trial Registration:*** ClinicalTrials.gov: NCT05552521 registered on the 20th of September 2022.

**Supplementary Information:**

The online version contains supplementary material available at 10.1186/s13613-025-01561-w.

## Background

Cardiovascular dysfunction is highly prevalent among patients with sepsis and septic shock [1], primarily driven by the release of cytokines, mitochondrial dysfunction, and tissue hypoxia, which subsequently contribute to myocardial injury [2, 3]. This impaired cardiovascular performance in septic patients, usually referred to as sepsis-induced myocardial dysfunction (SIMD, or septic cardiomyopathy), has not been adequately defined. SIMD may be very heterogeneous, manifesting as left ventricular (LV) or right ventricular (RV) dysfunction, impaired systolic performance and/or diastolic filling [4, 5]. Moreover, apart from such heterogeneity, the prevalence of progression and/or reversibility of SIMD is still not entirely clear due to the paucity of longitudinal studies [6]. Consequently, the full impact of SIMD on patient outcomes remains unclear. LV diastolic function (LVDD) has been previously associated with greater mortality [7, 8], but recent evidence has questioned this association [9] and the contribution of LV systolic dysfunction (LVSD) is also uncertain [10–12]. However, there are significant discrepancies in the literature, likely due to a considerable variability in the methodologies employed to investigate cardiac dysfunction within the context of a complex condition, such as sepsis. Whilst echocardiography is essential to characterize SIMD [13], conventional echocardiographic parameters are likely to suffer from the profound variations of loading conditions encountered in septic patients [14]. In this regard, speckle-tracking echocardiography (STE) has gained attention as a modality for detecting early myocardial alterations in sepsis that are not easily identifiable through conventional echocardiographic methods [15–17]. STE seems less influenced by preload conditions, though it is still dependent on ventricular loading [18, 19]. Hence, STE has been proposed to improve the detection of cardiac dysfunction in critically ill patients, a population that presents numerous challenges in the application of existing diagnostic guidelines [20].

In a longitudinal echocardiography study conducted in a population of septic shock patients, we aimed at characterizing the prevalence, evolution and prognosis of SIMD based on two different set of criteria, non-STE and STE-based. In particular, we hypothesized that a definition based on STE criteria would show a higher prevalence of SIMD and greater prognostic value as compared to a definition based on non-STE echocardiographic parameters.

## Methods

A single-center prospective observational study was conducted between October 2022 and December 2023 in the general intensive care unit (ICU) of Hospital Garcia de Orta, Almada (Portugal). The study was approved by the Hospital Garcia de Orta and Instituto de Medicina Molecular Ethics committees (T01/2022). Informed consent was signed by the patient or by the next of kin. The study was officially registered on ClinicalTrials.gov: NCT05552521 on September 20, 2022. Although the study’s primary outcome at the time of registration focused on LVDD and its evolution during the 30 days, the echocardiographic data collected within the research framework also encompassed thorough assessments of LV and RV systolic function since the beginning of the study. Consequently, we decided to report the study as a more comprehensive investigation concentrating on the overall incidence of SIMD rather than solely evaluating LVDD.

We included all adult (≥ 18-year-old) patients admitted consecutively to the ICU with septic shock, diagnosed as defined by the Surviving Sepsis Campaign criteria [21]: sepsis and (despite adequate volume resuscitation) the presence of persistent hypotension (requiring vasopressors to maintain mean arterial pressure – MAP - ≥ 65 mmHg) and lactate ≥ 2 mmol/L. Patients were excluded if any of the following was present: pregnancy, congenital heart disease, valve prosthesis, more than mild aortic or mitral disease, known previous cardiomyopathy with moderate to severe LVSD and/or RV systolic dysfunction (RVSD), chronic atrial fibrillation (AF), ischemic heart disease, acute pulmonary embolism, and inadequate image quality. Patients with new-onset AF were excluded only if the arrythmia was present during the timepoint of echocardiography evaluation.

Patients were treated according to the Surviving Sepsis Campaign Guidelines [21], and although the echocardiography results were not concealed from the treating physicians, therapy was not titrated to reach a specific echocardiographic goal.

### Echocardiography assessment and definition of SIMD

A comprehensive echocardiogram including STE parameters was performed at three predefined time-points: the earliest opportunity following admission (within the first 24 h), and on the 7th and 30th day after ICU admission (with a tolerance of ± 24 h according to the availability of the echocardiographer). All echocardiographic measurements were obtained using a Vivid IQ ultrasound machine (General Electric Healthcare ExcludedElement 1T, Chicago, Illinois, USA), with ECG-gating, at end-expiration, averaged from three measurements (since arrythmias were an exclusion criterion, no additional measurements were needed). Experienced operators on critical care echocardiography, certified or undergoing European Diploma in Advanced Critical Care EchoCardiography (EDEC) certification, acquired images and clips. These were analyzed offline and separately by one EDEC certified operator and two cardiologists with experience in critical care echocardiography. All operators assessing cardiac function were blinded to the patient’s outcome; also, they were blinded from each other. Images were analyzed using dedicated software (GE Echo PAC PC v206, GE HealthcareExcludedElement 3T, Chicago, Illinois, USA). Repeated measurements for different echocardiographic parameters were taken by the same physician, and two different physicians subsequently repeated the calculations to assess the inter- and intra-observer variability [22]. Echocardiographic data were reported according to the Preferred Reporting Items for Critical care Echocardiography Studies (PRICES) recommendation [23, 24], and PRICES checklists are reported in the supplementary materials.

We used two definitions of SIMD, obtained with either non-STE or STE echocardiography parameters. Regarding the use of non-STE parameters to diagnose SIMD, the chamber dimensions and the systolic function were assessed and classified according to the European Society of Cardiovascular Imaging (EACVI) and American Society of Echocardiography (ASE) guidelines [25]. LVDD was classified according to the 2016 ASE/EACVI guidelines [26]. For the STE imaging acquisition of the LV, RV and left atrium (LA), the operator maximized the frame rate per second (76 ± 24 fps) [27]. A higher frame rate was set when patients were tachycardic. Minimum strain quality criteria included: exclusion of a maximum of one segment per view, measurement of all three views (apical 4-, 2- and 3-chambers) for LV global longitudinal strain (GLS), apical 4-chambers for RV-GLS, and apical 4- and 2-chambers for peak atrial longitudinal strain (PALS). The software automatically tracked the endocardial contours throughout the cardiac cycle and manual adjustments were made to the contours as needed to optimize tracking. Classification of LV- and RV-GLS followed the consensus document of the EACVI/ASE/Industry Task Force [27]. The LA strain was used to assess reservoir function, using definitions from previous studies [28, 29]. From a practical perspective, we defined:

- LVSD as LV ejection fraction (EF) < 50% (non-STE) or LV-GLS >-15% (STE);
- LVDD according to 2016 ASE/EACVI guidelines (non-STE) [26] or PALS < 24% (STE);
- RV systolic dysfunction (RVSD) as tricuspid annular plane systolic excursion (TAPSE) < 17 mm (non-STE) or RV-GLS > – 5% (STE).

Regarding the definition of SIMD, we investigated the influence of using a broad definition in which any patient is diagnosed with SIMD if at least one of the three types of dysfunctions is present. We primarily aimed at studying the prevalence of SIMD using STE criteria vs. non-STE parameters, assessing also the evolution over the first week and finally at one month, and studying the prognostic impact of such broad definition of SIMD. In the longitudinal analysis, we made a post-hoc assumption before proceeding with statistical analysis: we assumed that SIMD developed (or remained present) in all non-survivors before their death. Indeed, all of them died in refractory shock that required a significant increase in the infusion of vasoactive drugs, indicating the likelihood of cardiovascular dysfunction. Recovery is defined in a dichotomous manner and intended as the restitution of all criteria used for LVSD, RVSD and LVDD to normal. Hence, any improvement (i.e. LVSD) that failed to reach the normal cut-off was still classified as SIMD.

Although echocardiographic parameters were reported according to the 2016 ASE/EACVI guidelines [25, 26] and with very good adherence to the PRICES recommendation (checklist in supplementary material) [24], cardiac dysfunction was defined as described in the previous paragraph.

### Clinical data and biochemical biomarkers

We collected demographics and clinical data. A search for previous cardiac function described by echocardiography was performed in the patient’s history through the national patient healthcare registry. Surprisingly, echocardiography data were present in all patients within the last year before admission, from ambulatory exams made as part of a routine healthcare check-up allowed by our national healthcare system. We used it to assess cardiac conditions related to exclusion criteria (such as moderate to severe left/right ventricular systolic function, valvular disease, etc.). In these reports, at least moderate LVSD and RVSD were classified by LVEF < 40% and TAPSE < 15 mm, respectively. Conversely, the reporting of LVDD was usually not performed thoroughly according to the current guidelines. None of these reports included STE data. Clinical severity was assessed by the Acute Physiology and Chronic Health Evaluation II (APACHE II), the Simplified Acute Physiology Score II (SAPS II), and the Sequential Organ Failure Assessment (SOFA). Acute kidney injury (AKI) was defined according to the Kidney Disease Improving Global Outcomes (KDIGO) guidelines [30]. Invasive arterial pressure was continuously monitored from an arterial line, and central venous pressure (CVP) was measured from a central venous catheter.

### Statistics

The distribution of quantitative variables was tested through the Kolmogorov-Smirnov test. Continuous variables were expressed as median and interquartile range and compared between groups using the non-parametric Mann-Whitney U-test. Categorical variables were expressed as numbers and proportions (%) and compared with Fisher’s exact test. All tests were two-tailed, and a p-value < 0.05 was considered statistically significant. Logistic regression was employed to analyze 30-day mortality as a binary outcome, estimating the odds of death, and Cox proportional hazards regression was used to analyze the time to death, providing a more detailed risk of mortality over the 30-day period. Data were right-censored as alive at the 30-days and those lost to follow-up before 30 days. Baseline (day 1) measurements of cardiac function (LVSD, RVSD, and LVDD) were included as fixed covariates in the model to assess their prognostic value for 30-day mortality. The longitudinal changes in these variables on days 7 and 30 were analyzed separately and not included as time-dependent covariates in the model. We used the Hosmer-Lemeshow test to evaluate goodness of fit and calibration for logistic regression models and partial residuals (Schoenfeld residuals) to assess the assumption of proportional hazards. Repeatability analysis was performed using an intraclass correlation coefficient with two different operators, blinded to each other’s results (Supplementary material). Statistical analysis was performed using IBM^®^ SPSS Statistics for Windows, version 29 (IBM^®^ Corp., Armonk, N.Y., USA).

## Results

**Fig. 1:**
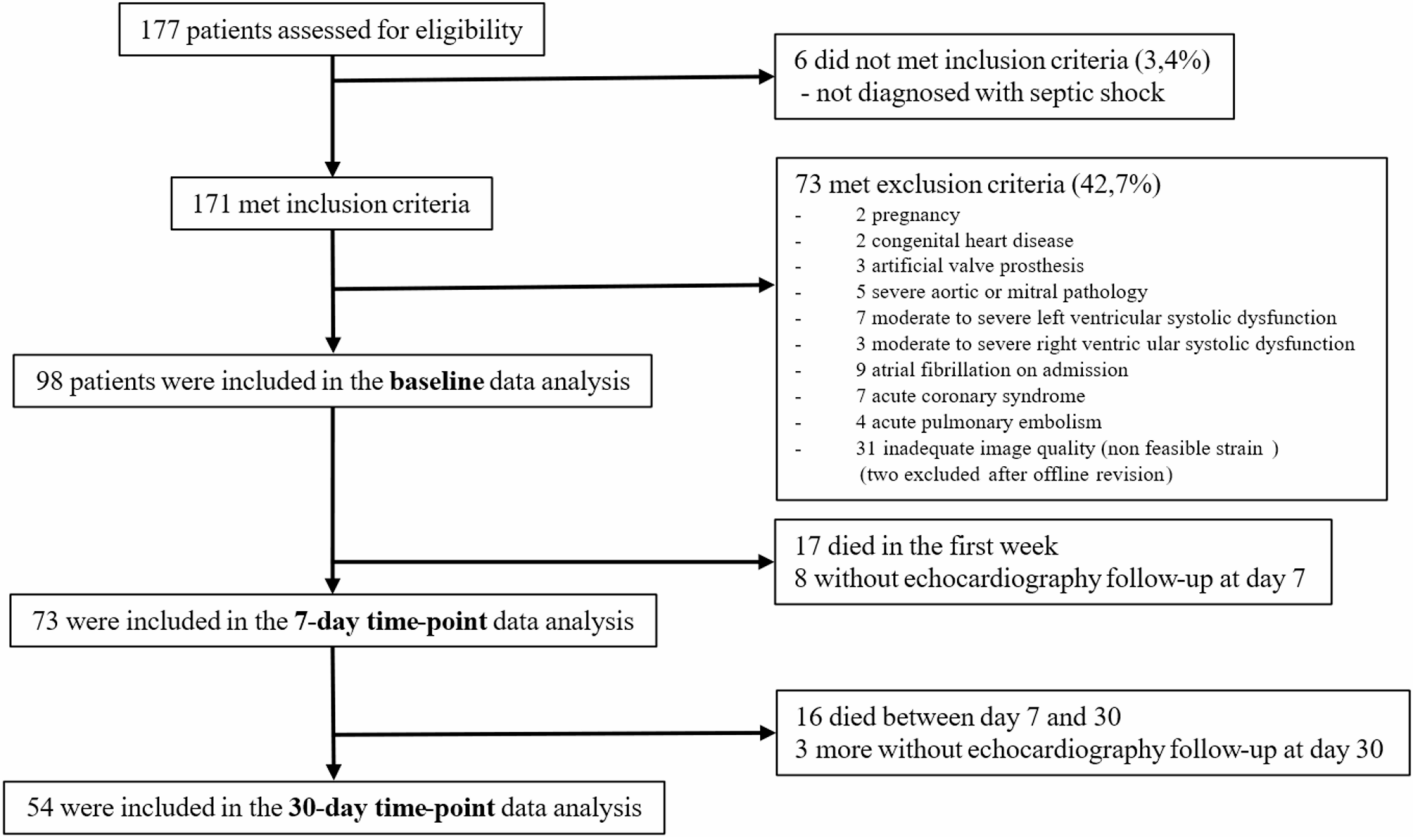
Flowchart for patients’ selection

As shown in Fig. 1, of the initial 177 eligible patients, 100 patients were included upon ICU admission. The flowchart describes the exclusions and subsequent availability of follow-up echocardiography for those included (Fig. 1). Of note, a complete STE exam on day 1 was performed in two patients who were subsequently excluded as only PALS data were judged appropriate on off-line calculation, while the LV- and RV-GLS were not. No patients had atrial fibrillation at the three timepoints of the study. Hence, a final population of 98 patients on day 1 was screened for SIMD with both criteria. Mortality at 30 days was 33.7% (*n* = 33/98). For the follow-up, 73 patients were available at 7 days (17 died and 8 echocardiograms were missing), and 54 patients analyzed at 30 days (further 16 died between day 7 and day 30, and 11 had missing follow-up).

### Baseline clinical characterization

Table 1 provides the baseline characteristics, severity scores, clinical outcomes, organ support and failure, and hemodynamics on day 1 in the overall population. We further provide the baseline data separated into the following three subgroups: no SIMD (*n* = 25), SIMD by STE only (*n* = 16), and SIMD by both STE and non-STE parameters (*n* = 54). Baseline data is not reported for the subgroup of patients with cardiac dysfunction by non-STE parameters only, as there were only three. Table 2 provides the echocardiographic data collected on day 1 for the overall population and the three above-mentioned subgroups. Of note, on review of premorbid echocardiogram, all of the included patients had normal LVEF and TAPSE, hence no patients had baseline mild LVSD/RVSD).

**Table 1:** Baseline characteristics, severity scores, clinical outcomes, organ support and hemodynamics in day 1 of echocardiography in the overall population, and distribution also according to subgroups with no septic-induced myocardial dysfunction (No SIMD), or SIMD by speckle tracking echocardiography (STE) only, or SIMD diagnosed by both STE and non-STE parameters

	All	No SIMD	SIMD by STE only	SIMD by STE and non-STE
	*n* = 98*	*n* = 25*	*n* = 16*	*n* = 54*
Demographics				
Age, years	64.5 [51.8–74.0]	60 [52–71]	62 [47–78]	68 [54–74]
NCTFemale,* n (%)*	42 (42.9)	12 (48.0)	9 (56.2)	20 (37.0)
Obesity	23 (23.5)	5 (20.0)	3 (18.8)	15 (27.8)
Hypertension, n (%)	56 (57.1)	12 (48.0)	9 (56.2)	36 (66.7)
DM, n (%)	35 (35.7)	9 (36.0)	7 (43.8)	19 (35.2)
CKD, n (%)	15 (15.3)	5 (20.0)	4 (25.0)	6 (11.1)
COPD, n (%)	14 (14.3)	3 (12.0)	3 (18.8)	8 (14.8)
Severity scores and outcomes				
APACHE II	20.0 [13.0–26.0]	17 [13–24]	16 [9–20]	22 [16–28]
SAPS II	44.0 [32.0-57.2]	42 [32–54]	38 [27–52]	48 [35–62]
7-day mortality, n (%)	17 (17.3)	3 (12.0)	1 (6.2)	12 (22.2)
30-day mortality, n (%)	33 (33.7)	6 (24.0)	3 (18.8)	23 (42.6)
Biomarkers				
hsTn, ng/mL	37 [20-165]	30 [15–41]	28 [14–64]	90 [29–270]
NT-proBNP, pg/mL	4584 [1342-12062]	2475 [1519–6502]	1689 [627–4172]	10,079 [2182–22006]
Lactate (arterial), mmol/L	4.1 [2.8–6.4]	2.9 [2.3–4.1]	3.7 [2.7–5.8]	4.9 [3.2–6.9]
ScvO2, %	74.0 [67.8–78.9]	74 [69–78]	78 [70–82]	73 [65–78]
Pv-a CO2	5.4 [4.6–7.6]	5 [4.2–6.8]	5.2 [5-7.9]	5.5 [4.6–8.6]
Source of sepsis				
Pulmonary, n (%)	37 (37.8)	10 (40.0)	6 (37.5)	21 (38.9)
Abdominal, n (%)	35 (35.7)	9 (36.0)	7 (43.8)	18 (33.3)
Soft tissue and skin, n (%)	10 (10.2)	3 (12.0)	0	7 (13.0)
Urologic, n (%)	6 (6.1)	1 (4.0)	1 (6.2)	3 (5.6)
Bacteremia, n (%)	6 (6.1)	1 (4.0)	0	4 (7.4)
Others, n (%)	4 (4.1)	1 (4.0)	2 (12.5)	1 (1.8)
Hemodynamics (at the time of the echocardiogram)				
Systolic AP, mmHg	108 [97.8-121.2]	115 [101–126]	112 [103–121]	104 [95–120]
Diastolic AP, mmHg	54 [48.0-60.2]	52 [47–55]	56 [48–71]	54 [49–61]
Mean AP, mmHg	72 [65.0-80.2]	75 [68–78]	76 [69–86]	70 [65–80]
Heart rate, bpm	94 [76.8-105.8]	87 [71–97]	88 [66–102]	99 [89–113]
CVP, mmHg	8 [4.5–12.0]	6 [4–9]	8 [4–12]	8 [5–13]
Cardiac index, L/min/m^2^	2.9 [2.2–3.9]	3.2 [2.4–3.8]	3.4 [2.5–4.4]	2.7 [2.2–3.5]
Fluid balance (24 h), mL	1369 [190.8-2064.8]	1320 [0-2025]	1416 [799–2165]	1400 [133–2083]
NE dose, mcg/kg/min	0.46 [0.20–0.88]	0.33 [0.2–0.9]	0.70 [0.20–0.88]	0.50 [0.20–0.96]
2nd vasopressor, n (%)	31 (31.6)	6 (24)	5 (31.3)	20 (37)
Inotrope, n (%)	3 (3.1)	1 (24)	0 (0)	2 (3.7)

*Data is not reported for the subgroup of patients that had cardiac dysfunction by non-STE parameters only, as these were only three patients

Data are presented as median [IQR] unless otherwise specified, IQR, interquartile range, Mann-Whitney U test for continuous variables and Fi7sher exact test for categorical variables

DM, Diabetes Mellitus; CKD, chronic kidney disease; COPD, chronic obstructive pulmonary disease; APACHE II, acute physiology and chronic health evaluation II; SAPS II, acute physiology and chronic health evaluation II; SOFA, sequential organ failure assessment; ICU, intensive care unit; LOS, length of stay; AKI, acute kidney injury; RRT, continuous renal replacement therapy; MVi, invasive mechanical ventilation; NE, norepinephrine; hsTn, high sensitivity troponin; NT-proBNP, N-terminal pro b-type natriuretic peptide; AP, arterial pressure; CVP, central venous pressure; Pv-a CO2, central venous-to-arterial carbon dioxide difference; SVRI, systemic vascular resistance indexed

**Table 2:** Echocardiographic parameters on day 1 in the overall population and distribution in subgroups with no septic-induced myocardial dysfunction (No SIMD), or SIMD by speckle tracking echocardiography (STE) only, or SIMD diagnosed by both STE and non-STE parameters

	All	No SIMD	SIMD by STE only	SIMD by STE and non-STE	
	*n* = 98	*n* = 25	*n* = 16	*n* = 54	p-value
Left ventricle					
Dimensions					
IVS, mm	9 [8–10]	10 [8–10]	9 [8–12]	9 [9–10]	0.864
LVPW, mm	9 [8–10]	9 [8–10]	10 [9–10]	9 [8–10]	0.522
LVEDV, mL	98 [82–109]	98 [86–120]	100 [74–111]	90 [84–105]	0.677
LVESV, mL	48 [37–60]	42 [34–54]	40 [31–50]	55 [46–62]	**< 0.001**
Function					
LV S´, cm/s	10 [8–13]	11 [10–12]	13 [11–15]	9 [7–11]	**< 0.001**
LVEF, %	51 [41–58]	58 [56–61]	58 [57–62]	41 [36–48]	**< 0.001**
LV-GLS, %	– 13.4 [– 17.6– – 11.1]	– 18 [– 20– – 17]	– 16 [– 19– – 11]	– 12 [– 13– – 10]	**< 0.001**
LVOT– VTI, cm	19 [15–24]	23 [19–26]	23 [19–26]	16 [13–19]	**< 0.001**
Left atrium					
E wave, cm/s	79 [67–90]	79 [69–97]	81 [68–84]	76 [62–91]	0.242
A wave, cm/s	56 [44–76]	62 [52–86]	57 [52–70]	49 [36–68]	**0.012**
Deceleration time, msec	163 [122–217]	183 [122–237]	183 [116–240]	156 [123–201]	0.404
E/A	1.3 [1.0– 1.7]	1.3 [0.9–1.6]	1.3 [1– 1.5]	1.4 [1– 2.3]	0.392
e’ lateral, cm/s	10 [8–13]	12 [9–15]	13 [8–17]	9 [7–12]	**0.034**
e’ septal, cm/s	8 [7–10]	9 [8–10]	10 [8–14]	8 [5–10]	**0.016**
E/e’	8.2 [6.4–9.9]	7.8 [6– 9.6]	6.3 [5.4–8.9]	8.6 [7.1–10.7]	**0.024**
LAVi, mL/m2	25 [19–32]	26 [21–34]	22 [20–30]	23 [16–32]	0.860
PALS, %	18 [12–24]	25 [20–34]	15 [12–20]	14 [10–20]	**< 0.001**
Right ventricle					
RV– GLS, %	– 16.3 [– 19.4 to – 13.6]	– 19 [– 22 to – 16]	– 18 [– 21 to – 15]	– 15 [– 17 to – 10]	**< 0.001**
TAPSE, mm	19 [16–21]	20 [19–22]	20 [18–21]	17 [14–20]	**< 0.001**
FAC, %	42 [36–48]	47 [42–53]	42 [41–47]	40 [32–46]	**< 0.001**
sPAP, mmHg	36 [26–43]	31 [26–46]	37 [28–46]	36 [22–40]	0.364

Data are presented as median [IQR] unless otherwise specified, IQR, interquartile range, Mann-Whitney U test for continuous variables and Fisher exact test for categorical variables

IVS, interventricular septum; LV, left ventricle; LVPW, LV posterior wall; LVEDD, LV end-diastolic diameter; LVESD, LV end-systolic diameter; LVEDV, LV end-diastolic volume; LVESV, LV end-systolic volume; LV S’, LV systolic myocardial velocity; LVEF, left ventricle ejection fraction; LV-GLS, LV global longitudinal strain; LVOT VTI, LV outflow tract velocity-time integral; E, peak early inflow velocity; A, peak late inflow velocity; E/A, peak early inflow velocity to peak late inflow velocity; e’, peak early longitudinal diastolic myocardial velocity; E/e’, peak early inflow velocity to peak early longitudinal diastolic myocardial velocity; LAVi, left atrial volume indexed; PALS, peak atrial longitudinal strain during the reservoir phase; RV, right ventricle; FAC, fractional area change; TAPSE, tricuspid annular plane systolic excursion; sPAP, systolic pulmonary artery pressure

### Prevalence of SIMD

**Fig. 2:**
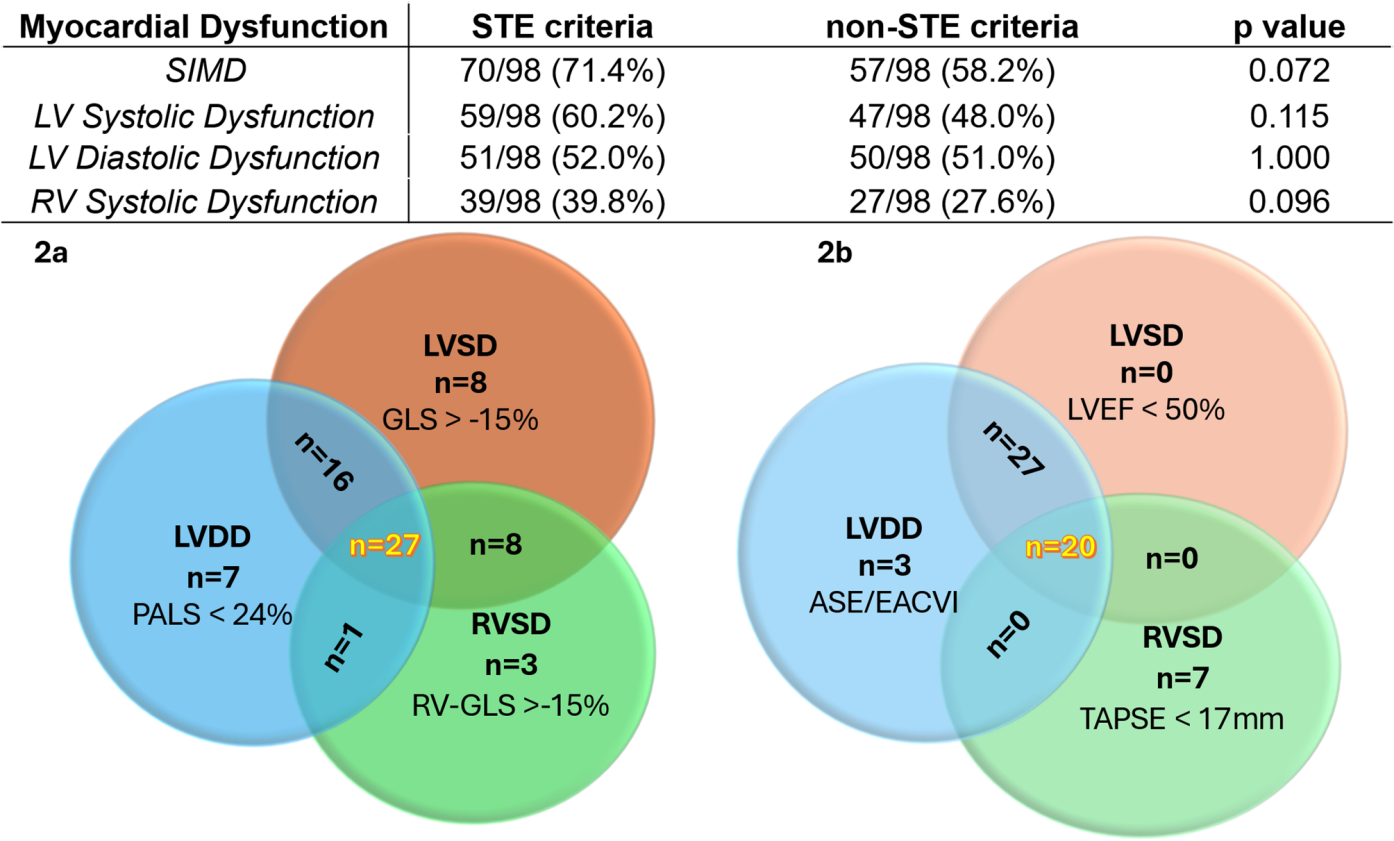
Venn diagrams describing the clinical spectrum and distribution of septic-induced myocardial dysfunction on day 1. Distribution is shown according to the speckle tracking echocardiography criteria (**a**) or to non-STE parameters (**b**). STE, speckle-tracking echocardiography; SIMD, sepsis-induced myocardial dysfunction; ASE/EACVI, American Society of Echocardiography / European Association of Cardio-Vascular Imaging guidelines; LV, left ventricle; SD, systolic dysfunction; DD, diastolic dysfunction; RV, right ventricle; GLS, global longitudinal strain; LVEF, LV ejection fraction; PALS, peak atrial longitudinal strain; TAPSE, tricuspid annular plane systolic excursion

When SIMD was defined as the presence of any myocardial dysfunction (LVSD and/or LVDD and/or RVSD), on day 1, it occurred in 70/98 patients using the STE criteria (71.4%), and in 57/98 patients using non-STE criteria (58.2%; *p* = 0.072). Figure 2 also shows the two Venn diagrams with the overall distribution and overlap of the three types of myocardial dysfunction on day 1, according to the STE (Fig. 2a) or non-STE approach (Fig. 2b). The LVSD had a non-significantly higher prevalence with STE criteria: *n* = 59/98 (60.2%) vs. *n* = 47/98 (48.0% using LVEF; *p* = 0.115). The RVSD was also not significantly higher when using STE: *n* = 39/98 (39.8%) compared to the non-STE approach with TAPSE (*n* = 27/98, 27.6%; *p* = 0.096). None of the patients with LVEF above 50% had LV-S’ wave below 7.5 cm/s, and none of the patients with TAPSE above 17 mm had FAC below 35%. Conversely, LVDD was diagnosed with almost identical prevalence: *n* = 51/98 (52.0% by STE with PALS) vs. *n* = 50/98 (51.0% by non-STE; *p* = 1.00). When LVDD was graded according to 2016 ASE/EACVI guidelines, we could identify: *n* = 32/50 patients with grade I (64%), *n* = 5/50 with grade II (10%) and *n* = 13/50 with grade III (26%).

### Evolution of SIMD

**Fig. 3:**
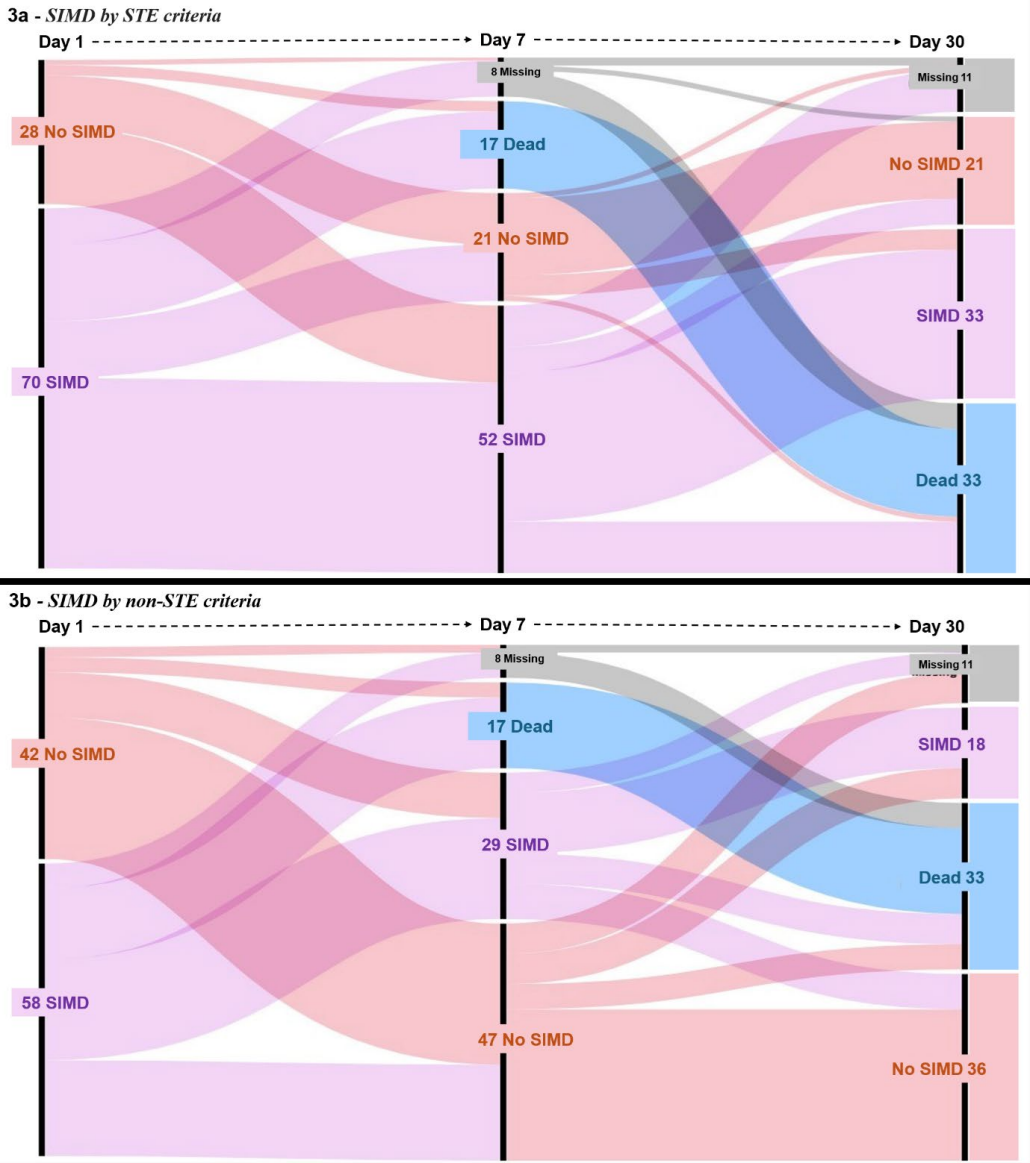
Alluvial diagrams showing the difference between the baseline incidence of sepsis-induced myocardial dysfunction (SIMD) by speckle tracking echocardiography (STE) (**a**) and by non-STE (**b**), and their respective evolution at days 7 and 30

The progression of SIMD from day 1 to day 7 and to day 30 using both STE and non-STE criteria is shown in the alluvial plots (Fig. 3a and b). Further details can be found in the supplementary material, where we describe the temporal changes, along with data on mortality. Overall, follow-up for both groups was not feasible in 8 and 11 patients on day 7 and day 30, respectively. The evolution of cardiac biomarkers and echocardiographic parameters on day 1, 7 and 30 is also available as supplementary material.

Looking at the longitudinal evolution, we assumed that SIMD remained present or developed in all non-survivors. Of the 70 patients diagnosed with SIMD by STE criteria on day 1, out of 63 patients completing adequate follow-up on day 7, only *n* = 11/63 had a recovery of cardiac function at this point (17.5%). By day 30, 64 of the 70 patients diagnosed with SIMD on day 1 completed echocardiographic follow-up (*n* = 6 missing); of these, only *n* = 12/64 had a recovery of cardiac function (18.8%). One echocardiogram was missing for the other 28 patients diagnosed with normal cardiac function on day 1 using STE. At follow-up on day 7, in *n* = 10/27 patients the diagnosis remained unchanged, whilst SIMD developed in *n* = 17/27 (63.0%) patients (15 with SIMD and two deceased). By day 30, follow-up was missing in 5 patients with normal function on day 1; in this subgroup, *n* = 14/23 developed SIMD (60.9%, 9 SIMD and 5 deceased), whilst *n* = 9/23 (39.1%) remained with normal function.

In the non-STE analysis, 57 patients identified with SIMD on day 1, 51 and 53 had follow-up echocardiograms on day 7 and day 30, respectively. Assuming that SIMD remained present/developed in non-survivors, *n* = 18/51 patients recovered cardiac function by day 7 (35.3%) and *n* = 13/53 recovered normal function by day 30 (24.5%). For the 41 patients with normal function by non-STE, 2 and 7 follow-ups were missing on day 7 and 30, respectively. Progression to SIMD by day 7 happened in *n* = 11/39 patients (28.2%, 8 SIMD and 3 deceased). By day 30, *n* = 11/34 patients had progression to SIMD (32.4%, 5 SIMD and 6 deceased), whilst *n* = 23/34 (67.6%) remained with normal function.

The recovery rate at day 7 from the initial diagnosis of SIMD on day 1 was significantly higher when evaluated by non-STE: *n* = 18/51 (35.3%) vs. *n* = 11/63 (17.5%, in STE *p* = 0.033), whilst the recovery at day 30 was not significantly different: *n* = 13/53 (24.5%, in non-STE; *p* = 0.501) vs. *n* = 12/64 (18.8%, in STE). In those diagnosed with normal function on day 1, the progression to SIMD was significantly higher with STE method at day 7 (*n* = 17/27 vs. *n* = 11/39 with non-STE; *p* = 0.0006). By day 30, the progression from normal function on day 1 to SIMD was not significantly higher with STE (*n* = 14/23 vs. *n* = 11/34 with non-STE; *p* = 0.056).

In our cohort, 12 patients were always diagnosed with SIMD with STE from day 1 to day 30 (*n* = 12/70, 17.1%). This prevalence was not significantly higher (*n* = 18/57, 31.6%) in those classified with non-STE criteria (*p* = 0.063). The occurrence of “only late SIMD” (normal function on day 1 and day 7, SIMD on day 30) was uncommon (*n* = 2/98, with both STE and non-STE criteria).

### Prognostic value of SIMD

Looking at the 30-day mortality (*n* = 33), when using STE criteria, we found a not significantly higher mortality in patients diagnosed with SIMD on day 1 (*n* = 28/70, 40%) as compared to those with normal cardiac function (*n* = 5/28, 17.8%; *p* = 0.057). There was a significantly higher mortality at 30 days in patients diagnosed with SIMD with non-STE criteria on day 1 (27/57, 47.4% vs. 6/41, 14.6% respectively; *p* = 0.001).

When analyzing subgroups according to cardiac dysfunction, we found a significant association between LVSD, as defined by STE criteria, and mortality (*n* = 25/59, 42.4% vs. *n* = 8/39, 20.5% in normal LV systolic function, *p* = 0.030). However, no association was observed when LVEF was used as the criterion to identify LVSD (*n* = 20/47 vs. *n* = 13/51 in normal LV systolic function, *p* = 0.089). Conversely, we identified a significant association between LVDD according to 2016 ASE/EACVI guidelines and mortality (*n* = 23/50 vs. *n* = 10/48 in normal LV diastolic function, *p* = 0.011), while the association was not significant when using STE criteria (*n* = 22/51 vs. *n* = 11/47 in normal LV diastolic function, *p* = 0.054). In the subgroup of patients diagnosed with RVSD by STE, 30-day mortality did not differ (*n* = 14/39, 35.9% vs. *n* = 19/59, 32.2% in normal RV systolic function; *p* = 0.828). Similarly, we found no differences when RVSD was diagnosed using non-STE (*n* = 12/27, 44.4% vs. *n* = 21/71, 29.6% in normal RV systolic function; *p* = 0.231).

Although LVSD and LVDD (but not RVSD) with diagnosis made by STE or non-STE were associated with 30-day mortality in a univariable logistic regression, neither type of dysfunction remained significant in the multivariable logistic regression (supplementary material). In a multivariable Cox regression model (Table 4), including non-STE and STE criteria, only the occurrence of LVDD diagnosed using 2016 ASE/EACVI criteria significantly predicted 30-day mortality, with an hazard ratio of 4.6 (95% CI 1.6–13.6, *p* = 0.005).

**Table 4:** Cox regression coefficients and hazard ratio (HR) showing the likelihood of 30-day mortality on septic shock patients according to speckle tracking echocardiography (STE) and non-STE based criteria for septic induced myocardial dysfunction (SIMD)

Variables	Cox regression						HR 95% Confidence Interval		
	Coefficient	SE	χ2		*p*-value	HR			
							Lower Bound		Upper Bound
LVEF, %	0,014	0,029	0,223		0,637	1,014	0,958		1,072
LV-GLS; %	0,053	0,090	0,352		0,553	1,055	0,885		1,257
**LVDD-ASE/EACVI**	**1**,**535**	**0**,**547**	**7**,**870**		**0**,**005**	**4**,**643**	**1**,**588**		**13**,**572**
PALS, %	-0,004	0,026	0,029		0,865	0,996	0,945		1,048
TAPSE, mm	-0,072	0,053	1,836		0,175	0,931	0,839		1,033
RV-GLS, %	0,001	0,076	0,000		0,984	1,002	0,863		1,162
**Model**	***-2 Log likelihood***			***Chi-square***				***p-value***	
**summary**	225,771			14,979				0,020	

SE, standard error; LVEF, left ventricle ejection fraction; LV-GLS, left ventricle global longitudinal strain; LVDD-ASE/EACVI, left ventricle diastolic dysfunction according to American Society of Echocardiography and European Association of Cardiovascular Imaging; PALS, peak atrial longitudinal strain; TAPSE, tricuspid annular plane systolic excursion; RV-GLS, right ventricle global longitudinal strain

### Post-hoc analyses

According to the Venn diagram in Fig. 2, there was an important overlap of different types of cardiac dysfunction, which was more pronounced with non-STE, in particular because of the concomitant diagnosis of LVDD in all patients diagnosed with LVSD by means of reduced LVEF. Only 18 patients had a single cardiac dysfunction in the STE approach, and only 10 with the non-STE criteria. In a post-hoc analysis, we found no difference in 30-day mortality between these patients with single as compared to those with at least two dysfunctions. Only one patient had hyperdynamic LV systolic function (defined as LVEF > 70% [31]).

### Repeatability analysis

Intra- and inter-observer variability was calculated as the intraclass correlation coefficient (ICC) for LVEF, LV-GLS, peak early inflow velocity to peak late inflow velocity (E/A), septal peak early longitudinal diastolic myocardial velocity (e’), peak early inflow velocity to peak early longitudinal diastolic myocardial velocity (E/e’), PALS, RV-GLS, TAPSE and systolic pulmonary artery pressure (sPAP). Intra- and inter-observer variability ranged from 0.86 (95% CI 0.136–0.959) to 0.997 (95% CI 0.958–0.999) and from 0.807 (95% CI -0.007-0.956) to 0.983 (95% CI 0.256.0.996), respectively, with lower ICC for LV-GLS (Supplementary material).

## Discussion

We longitudinally investigated SIMD with echocardiography through STE and non-STE approaches, a controversial subject due to the lack of a consensus on its definitions. We identified three principal findings according to the prevalence of SIMD, its recovery or progression over the first 30 days, and its prognostic value. These findings can be summarized as follows: (1) there was a non-significant increase in SIMD prevalence on day 1 when diagnosed using STE criteria as opposed to non-STE parameters (*p* = 0.072); (2) the recovery rate from SIMD diagnosed on day 1 was significantly lower at day 7 if using STE approach, though the recovery by 30 days was comparable between methods; (3) adopting broad STE criteria (any LV and/or RV dysfunction) for the definition of SIMD demonstrated no additional prognostic value (*p* = 0.057) when compared with a non-STE approach (*p* = 0.001).

### Prevalence of SIMD

The first aim of our longitudinal study was to investigate how STE assessment influences the diagnosis of SIMD if used with very broad criteria (any LV or RV dysfunction). With such an approach, SIMD was diagnosed in almost three-quarters (71.4%) of septic shock patients, in contrast to a slightly lower but still high prevalence of SIMD (58.2%) when using non-STE criteria (roughly a 20% relative increase with STE). Prior research has reported a highly variable prevalence of cardiac dysfunction, ranging from 10 to 70% [4]. A recent systematic review and meta-analysis indicated that the prevalence of new-onset LVSD was approximately 20% [1]. Our study found a much higher prevalence of LVSD using STE (60.2%) with roughly a 25% relative increase from data gathered with LVEF (48.0%). Dalla et al. [16] reported that half of the septic patients with LVEF greater than 50% exhibited an abnormal LV-GLS (defined as >-15%), confirming the ability of GLS to identify early deterioration in LV performances. Regarding the incidence of LV and RV dysfunction, Orde et al. [15] identified a nearly two-fold prevalence when using strain techniques as compared to non-STE. Indeed, they reported a prevalence of 69% and 72% for LV and RV dysfunction, respectively. Of note, the comparability of their findings with ours is limited by the use of more liberal cut-off values employed in their study to define LV and RV dysfunction (RV-GLS >-21% and LV-GLS >-17%). Several factors may account for the high prevalence of SIMD observed in our cohort, which can be summarized as follows: the use of a broad SIMD definition, the inclusion of patients with septic shock with relatively high severity scores (several studies enrolled also patients with less severe septic disease), and the possibility that patients had LVDD already at baseline (not detected by the premorbid echocardiogram).

### Evolution and reversibility of SIMD

The second focus of our research was the progression of cardiac dysfunction at the 7- and 30-day follow-up. The progression of SIMD showed a recovery rate by day 30 of only about one-quarter (in STE) to one-fifth (in non-STE) of patients, with no differences between methods. However, among patients diagnosed with SIMD on day 1, cardiac function recovery was significantly more frequent at day 7 when evaluated using non-STE (35.3%) as compared to STE criteria (17.5%). Of note, this result might be biased from presuming that all non-survivors either retained or developed SIMD, possibly resulting in lower recovery estimates. Nonetheless, it appears that STE can still identify certain cardiac abnormalities in the first week that non-STE may initially overlook. Similarly, the STE identified a greater number of progressions from normal cardiac function on day 1 to SIMD on day 7 (*p* = 0.006), with also a trend towards more frequent progression to SIMD at day 30 (*p* = 0.056). Therefore, STE could be beneficial not only in recognizing early myocardial dysfunction but also during follow-up, monitoring both the recovery of cardiac function from SIMD or the development of SIMD in previously diagnosed normal cardiac function. The latter finding can be explained by at least two factors. First, some patients had not yet developed SIMD in the first 24 h (initial echocardiographic assessment). Second, since the STE method tends to detect SIMD with greater prevalence, it will identify a higher proportion of patients with dysfunction across all time points.

These results are not entirely surprising considering findings from other studies. For instance, Canesso et al. [32] found that values of LV- and RV-GLS recorded on day 1 increased significantly in survivors at day 7, but not in patients who died afterwards. In the same direction, Ng et al. [17] demonstrated significant improvements in LV-GLS in patients weaned off from vasoactive support. However, the course of cardiac dysfunction using non-STE and STE criteria has been reported with conflicting findings. Bazalgette et al. [33] reported a significant improvement in LV-GLS between the third and fifth day, whereas LVEF remained stable over time. Conversely, De Geer et al. described significant improvement in LVEF during a 7-day study period but not in LV-GLS [34].

### Prognostic implications of using strain vs. non-STE criteria for SIMD diagnosis

Finally, our findings indicate that 30-day mortality is higher in patients diagnosed with SIMD on day 1 via non-STE criteria (46.6% vs. no-SIMD 14.3%, *p* = 0.001). In contrast, the prognostic impact of SIMD diagnosed with STE appears more uncertain (40% vs. no-SIMD 17.9%, *p* = 0.057). Several previous studies have suggested a prognostic role for STE in critically ill septic patients. An altered GLS value upon admission, specifically greater than − 13%, has been identified as an independent predictor of mortality in septic shock [35, 36]. Moreover, significantly worse GLS values in septic non-survivors have been confirmed by two meta-analyses [37, 38]. Furthermore, most meta-analyses performed to date have not clearly identified a correlation between LVEF and mortality in sepsis [7, 11, 39]. A number of possible reasons may explain these different results. Interestingly, the most striking prognostic difference between STE and non-STE approaches was found for the impact of LVDD diagnosis using 2016 ASE/EACVI guidelines [26], whilst use of PALS did not seem to confer advantages. Nevertheless, PALS has just been introduced in the most recent guidelines for the assessment of LV diastolic function [40]. It is possible that in our study several patients had an already abnormal PALS at baseline before developing septic shock, hence decreasing its prognostic role. Whether PALS could help to identify early diastolic deterioration in ICU patients remains to be established. Another consideration on the prognostic role of LVDD seen in our study is the large overlap of LVSD and LVDD, with only 3 patients having purely LVDD. Hence, the increased mortality associated with non-STE assessment of LVDD should be mostly attributed to the coexistence of LVSD. Of note, a recent multicenter study [9] showed a very high prevalence of LVDD assessed by non-STE methods (76% vs. 51% in our cohort). However, in conflict with previous evidence [7, 8], the French study found no impact of LVDD on mortality. Several considerations may explain this difference, as the French study diagnosed LVDD if it was reported at least once in three echocardiograms performed over the first 3 days in a population with a mean age of 63 years (with almost half suffering from hypertension). The lack of association of LVDD with mortality in this study as compared to previous literature highlights the complexity and multifactorial interaction of sepsis, cardiac dysfunction and mortality, suggesting that targeted interventions may improve the outcome [41]. Indeed, in the French study [9] clinicians were not blinded from the echocardiogram findings, and patients with LVDD had a less positive fluid balance, which may suggest that awareness of LVDD had an impact on cautious fluid management.

### Limitations

This study presents several limitations. First, despite the relatively large sample size, it is a single-center investigation that requires external validation, and it could be underpowered for some associations. Indeed, the study was registered with the idea of evaluating LVDD progression but ended up in a more comprehensive assessment of SIMD. Second, we assumed that all deceased patients developed SIMD. While this is an approximation, the rationale for considering any deceased patient as maintaining or developing cardiac dysfunction was the development of refractory shock with escalating doses of vasoactive agents in all the 33 deceased patients. Moreover, the “lack of recovery” in a high percentage of survivors is likely influenced by the strict definition of recovery (normalization of all parameters), the severity of septic shock and the possibility of pre-existing LVDD. Third, we lost some patients to follow-up, introducing a degree of imprecision. Fourth, our cohort is not entirely representative of the general population, as we were able to exclude patients with known cardiomyopathy or LV and/or RV systolic dysfunction. Fifth, the accuracy of STE measurements is contingent upon interobserver variability, particularly in recognizing difficult endocardial borders. The quality of the images was not always optimal, as commonly happens in the ICU. For this reason, some patients had lower frame-per-second rates in the strain analysis, though it remained within the recommended range and was adjusted for heart rate [27]. The overall feasibility of STE in our population may seem high (76%), but it is in line with recent literature [42, 43]. In particular, it was positively influenced by a third of the population not invasively ventilated (feasibility: *n* = 36/38 vs. 62/91 for invasively ventilated patients). Sixth, the diagnosis of SIMD is dependent on the chosen cut-offs. A cut-off of -20% for RV-GLS as recommended in the guidelines [44] would have significantly increased the prevalence of RVSD from 39.8% (*n* = 39/98) to 83.7% (*n* = 82/98), with a significant difference vs. non-STE (*n* = 27/98). However, recent evidence suggests that the lower limit of normal value of GLS in the healthy population is around − 15% [45, 46]. Hence, using a cut-off of -20% could falsely diagnose SIMD in a not negligible proportion of patients. Another consideration is that the latest recommendations [47] suggest RV-FWS as the preferred indicator of RV function instead of RV-GLS. Nevertheless, the guidelines were published after our analysis started and we had no resources to change this in due course. Despite only 3% of patients received inotropes, it is important to note that most patients did not exhibit a profound depression of LV/RV contractility (median LVEF 51%, IQR 41–58%, and median TAPSE 19 mm, IQR 16–21 mm). Furthermore, it is crucial to emphasize that when reporting the number of patients receiving inotropic agents, we are describing the therapy administered at the time of the echocardiographic assessment, in accordance with the PRISCES statement. This reporting does not account for potential subsequent changes in therapy initiated after the echocardiogram. Finally, to minimize biases we enrolled all consecutive adult patients meeting the inclusion criteria, the operators performing the offline echocardiography analysis were blinded to the clinical outcomes, we used pre-specified definitions for SIMD, echocardiographic data acquisition was standardized, and images were acquired by experienced and certified operators. We also included a repeatability analysis, calculating intra- and inter-observer variability, and transparently acknowledged the missing follow-up data at different time points.

## Conclusions

When using broad criteria to diagnose SIMD, its prevalence in patients admitted to the ICU with septic shock was very high on day 1, especially when the STE criteria were applied. By day 30, the reversibility of SIMD was similar for both STE and non-STE echocardiographic assessments. A broad definition for diagnosing SIMD using STE did not appear to demonstrate a prognostic role, unlike SIMD diagnosed with non-STE parameters. While confirming the ability of STE to detect early cardiac dysfunction, this study also highlights the challenges of achieving a definition of SIMD diagnosis with prognostic value. However, these results need further validation with a larger sample size and a multicentric design to evaluate the significance of broad diagnostic criteria for SIMD.

## Supplementary Information

Below is the link to the electronic supplementary material.

Supplementary Material 1.

Supplementary Material 2.

Supplementary Material 3.

Supplementary Material 4.

Supplementary Material 5.

## Data Availability

Data and material supporting this project are not shared; it is an ongoing database construction for other projects. Registered at ClinicalTrials.gov: NCT05552521 on the 20th of September 2022.

## References

[1] Hasegawa D, Ishisaka Y, Maeda T, Prasitlumkum N, Nishida K, Dugar S, Sato R. Prevalence and prognosis of Sepsis-Induced cardiomyopathy: A systematic review and Meta-Analysis. J Intensive Care Med. 2023;38:797–808. 10.1177/08850666231180526. 37272081 10.1177/08850666231180526

[2] Hollenberg SM, Singer M. Pathophysiology of Sepsis-Induced cardiomyopathy. Nat Rev Cardiol. 2021;18:424–34. 10.1038/s41569-020-00492-2. 33473203 10.1038/s41569-020-00492-2

[3] Kakihana Y, Ito T, Nakahara M, Yamaguchi K, Yasuda T. Sepsis-Induced myocardial dysfunction: pathophysiology and management. J Intensive Care 2016. 10.1186/s40560-016-0148-1 PMC4804632 27011791

[4] Beesley SJ, Weber G, Sarge T, Nikravan S, Grissom CK, Lanspa MJ, Shahul S, Brown SM. Septic cardiomyopathy. Crit Care Med. 2018;46:625–34. 10.1097/CCM.0000000000002851. 29227368 10.1097/CCM.0000000000002851

[5] Vieillard-Baron A, Septic, Cardiomyopathy. Ann Intensive Care. 2011;1:6. 10.1186/2110-5820-1-6. 21906334 10.1186/2110-5820-1-6 PMC3159902

[6] Sanfilippo F, Orde S, Oliveri F, Scolletta S, Astuto M. The challenging diagnosis of septic cardiomyopathy. Chest. 2019;156:635–6. 10.1016/j.chest.2019.04.136. 31511158 10.1016/j.chest.2019.04.136

[7] Sanfilippo F, Corredor C, Fletcher N, Landesberg G, Benedetto U, Foex P, Cecconi M. Diastolic dysfunction and mortality in septic patients: A systematic review and Meta-Analysis. Intensive Care Med. 2015;41:1004–13. 10.1007/s00134-015-3748-7. 25800584 10.1007/s00134-015-3748-7

[8] Sanfilippo F, Corredor C, Arcadipane A, Landesberg G, Vieillard-Baron A, Cecconi M, Fletcher N. Tissue doppler assessment of diastolic function and relationship with mortality in critically ill septic patients: A systematic review and Meta-Analysis. Br J Anaesth. 2017;119:583–94. 10.1093/bja/aex254. 29121301 10.1093/bja/aex254

[9] Vignon P, Charron C, Legras A, Musset F, Slama M, Prat G, Silva S, Vandroux D, Müller G, Levy B, et al. Left ventricular diastolic dysfunction is prevalent but not associated with mortality in patients with septic shock. Intensive Care Med. 2025;51:94–105. 10.1007/s00134-024-07748-2. 39774865 10.1007/s00134-024-07748-2

[10] Sanfilippo F, Huang S, Messina A, Franchi F, Oliveri F, Vieillard-Baron A, Cecconi M, Astuto M. Systolic dysfunction as evaluated by tissue doppler imaging echocardiography and mortality in septic patients: A systematic review and Meta-Analysis. J Crit Care. 2021;62:256–64. 10.1016/j.jcrc.2020.12.026. 33461118 10.1016/j.jcrc.2020.12.026

[11] Huang SJ, Nalos M, McLean AS. Is early ventricular dysfunction or dilatation associated with lower mortality rate in adult severe sepsis and septic shock?? A Meta-Analysis. Crit Care. 2013;17:R96. 10.1186/cc12741. 23706109 10.1186/cc12741 PMC4056117

[12] Dugar S, Sato R, Chawla S, You JY, Wang X, Grimm R, Collier P, Lanspa M, Duggal A. Is left ventricular systolic dysfunction associated with increased mortality among patients with sepsis and septic shock?? Chest. 2023. 10.1016/j.chest.2023.01.010. 36646415 10.1016/j.chest.2023.01.010

[13] Vieillard-Baron A, Millington SJ, Sanfilippo F, Chew M, Diaz-Gomez J, McLean A, Pinsky MR, Pulido J, Mayo P, Fletcher N. A decade of progress in critical care echocardiography: A narrative review. Intensive Care Med. 2019;45:770–88. 10.1007/s00134-019-05604-2. 30911808 10.1007/s00134-019-05604-2

[14] Boissier F, Razazi K, Seemann A, Bedet A, Thille AW, de Prost N, Lim P, Brun-Buisson C. Mekontso dessap, A. Left ventricular systolic dysfunction during septic shock: the role of loading conditions. Intensive Care Med. 2017;43:633–42. 10.1007/S00134-017-4698-Z. 28204860 10.1007/s00134-017-4698-z

[15] Orde SR, Pulido JN, Masaki M, Gillespie S, Spoon JN, Kane GC, Oh JK. Outcome prediction in sepsis: speckle tracking echocardiography based assessment of myocardial function. Crit Care. 2014;18. 10.1186/cc13987. 10.1186/cc13987 PMC4227017 25015102

[16] Dalla K, Hallman C, Bech-Hanssen O, Haney M, Ricksten S-E. Strain echocardiography identifies impaired longitudinal systolic function in patients with septic shock and preserved ejection fraction. Cardiovasc Ultrasound. 2015;13:30. 10.1186/s12947-015-0025-4. 26134971 10.1186/s12947-015-0025-4 PMC4487964

[17] Ng PY, Sin WC, Ng AKY, Chan WM. Speckle tracking echocardiography in patients with septic shock: A case control study (SPECKSS). Crit Care. 2016;20. 10.1186/s13054-016-1327-0. 10.1186/s13054-016-1327-0 PMC4867983 27177587

[18] Choi JO, Shin DH, Cho SW, Song Y, Bin; Kim JH, Kim YG, Lee SC, Park SW. Effect of preload on left ventricular longitudinal strain by 2D speckle tracking. Echocardiography. 2008;25:873–9. 10.1111/J.1540-8175.2008.00707.X. 18986415 10.1111/j.1540-8175.2008.00707.x

[19] Burns AT, La Gerche A, D’hooge J, Macisaac AI, Prior DL. Left ventricular strain and strain rate: characterization of the effect of load in human subjects. Eur J Echocardiogr. 2010;11:283–9. 10.1093/EJECHOCARD/JEP214. 20026455 10.1093/ejechocard/jep214

[20] Gonzalez FA, Santonocito C, Maybauer MO, Lopes LR, Almeida AG, Sanfilippo F. Diastology in the intensive care unit: challenges for the assessment and future directions. Echocardiography. 2024;41. 10.1111/ECHO.15773. 10.1111/echo.15773 38380688

[21] Evans L, Rhodes A, Alhazzani W, Antonelli M, Coopersmith CM, French C, Machado FR, Mcintyre L, Ostermann M, Prescott HC, et al. Surviving sepsis campaign: international guidelines for management of sepsis and septic shock 2021. Intensive Care Med. 2021;47:1181–247. 10.1007/S00134-021-06506-Y. 34599691 10.1007/s00134-021-06506-y PMC8486643

[22] Bunting KV, Steeds RP, Slater K, Rogers JK, Gkoutos GV, Kotecha D. A practical guide to assess the reproducibility of echocardiographic measurements. J Am Soc Echocardiogr. 2019;32:1505–15. 10.1016/J.ECHO.2019.08.015. 31653530 10.1016/j.echo.2019.08.015

[23] Huang S, Sanfilippo F, Herpain A, Balik M, Chew M, Clau-Terré F, Corredor C, De Backer D, Fletcher N, Geri G, et al. Systematic review and literature appraisal on methodology of conducting and reporting Critical-Care echocardiography studies: A report from the European society of intensive care medicine PRICES expert panel. Ann Intensive Care. 2020;10. 10.1186/S13613-020-00662-Y. 10.1186/s13613-020-00662-y PMC7183522 32335780

[24] Sanfilippo F, Huang S, Herpain A, Balik M, Chew MS, Clau-Terré F, Corredor C, De Backer D, Fletcher N, Geri G, et al. The PRICES statement: an ESICM expert consensus on methodology for conducting and reporting critical care echocardiography research studies. Intensive Care Med. 2021;47:1–13. 10.1007/S00134-020-06262-5. 33275163 10.1007/s00134-020-06262-5

[25] Lang RM, Badano LP, Mor-Avi V, Afilalo J, Armstrong A, Ernande L, Flachskampf FA, Foster E, Goldstein SA, Kuznetsova T, et al. Recommendations for cardiac chamber quantification by echocardiography in adults: an update from the American society of echocardiography and the European association of cardiovascular imaging. J Am Soc Echocardiogr. 2015;28:1–e3914. 10.1016/j.echo.2014.10.003. 25559473 10.1016/j.echo.2014.10.003

[26] Nagueh SF, Smiseth OA, Appleton CP, Byrd BF 3, Dokainish H, Edvardsen T, Flachskampf FA, Gillebert TC, Klein AL, Lancellotti P, et al. Recommendations for the evaluation of left ventricular diastolic function by echocardiography: an update from the American society of echocardiography and the European association of cardiovascular imaging. J Am Soc Echocardiogr. 2016;29:277–314. 10.1016/j.echo.2016.01.011. 27037982 10.1016/j.echo.2016.01.011

[27] Voigt JU, Pedrizzetti G, Lysyansky P, Marwick TH, Houle H, Baumann R, Pedri S, Ito Y, Abe Y, Metz S, et al. Definitions for a common standard for 2D speckle tracking echocardiography: consensus document of the eacvi/ase/industry task force to standardize deformation imaging. Eur Heart J Cardiovasc Imaging. 2015;16:1–11. 10.1093/ehjci/jeu184. 25525063 10.1093/ehjci/jeu184

[28] Singh A, Addetia K, Maffessanti F, Mor-Avi V, Lang RM. LA strain for categorization of LV diastolic dysfunction. JACC Cardiovasc Imaging. 2017;10:735–43. 10.1016/j.jcmg.2016.08.014. 28017389 10.1016/j.jcmg.2016.08.014 PMC5741456

[29] Krittanawong C, Maitra NS, Hassan Virk HU, Farrell A, Hamzeh I, Arya B, Pressman GS, Wang Z, Marwick TH. Normal ranges of right atrial strain: A systematic review and Meta-Analysis. JACC Cardiovasc Imaging. 2023;16:282–94. 10.1016/J.JCMG.2022.06.022. 36648033 10.1016/j.jcmg.2022.06.022

[30] Khwaja AKDIGO. Clinical practice guidelines for acute kidney injury. Nephron Clin Pract. 2012;120. 10.1159/000339789. 10.1159/000339789 22890468

[31] Sato R, Sanfilippo F, Hasegawa D, Prasitlumkum N, Duggal A, Dugar S. Prevalence and prognosis of hyperdynamic left ventricular systolic function in septic patients: A systematic review and Meta-Analysis. Ann Intensive Care. 2024;14:1–10. 10.1186/S13613-024-01255-9/FIGURES/4. 38308701 10.1186/s13613-024-01255-9 PMC10838258

[32] de Canesso BLC, Borges M, de Deus Queiroz Santos IN, Ris TA, de Barros TH, Nobre MVL, Nunes V. Value of Speckle-Tracking echocardiography changes in monitoring myocardial dysfunction during treatment of sepsis: potential prognostic implications. Int J Cardiovasc Imaging. 2019;35:855–9. 10.1007/S10554-018-01525-1. 30847658 10.1007/s10554-018-01525-1

[33] Bazalgette F, Roger C, Louart B, Daurat A, Bobbia X, Lefrant JY, Muller L. Prognostic value and time course evolution left ventricular global longitudinal strain in septic shock: an exploratory prospective study. J Clin Monit Comput. 2021;35:1501–10. 10.1007/S10877-020-00620-W. 33216237 10.1007/s10877-020-00620-w

[34] De Geer L, Engvall J, Oscarsson A. Strain echocardiography in septic Shock - a comparison with systolic and diastolic function parameters, cardiac biomarkers and outcome. Crit Care. 2015;19:1–9. 10.1186/s13054-015-0857-1. 25882600 10.1186/s13054-015-0857-1 PMC4374340

[35] Chang WT, Lee WTWH, Lee WTWH, Chen PS, Su YR, Liu PY, Liu YW, Tsai WC. Left ventricular global longitudinal strain is independently associated with mortality in septic shock patients. Intensive Care Med. 2015;41:1791–9. 10.1007/s00134-015-3970-3. 26183489 10.1007/s00134-015-3970-3

[36] Innocenti F, Palmieri V, Guzzo A, Stefanone VT, Donnini C, Pini RSOFA. Score and left ventricular systolic function as predictors of Short-Term outcome in patients with sepsis. Intern Emerg Med. 2018;13:51–8. 10.1007/S11739-016-1579-3. 27909859 10.1007/s11739-016-1579-3

[37] Sanfilippo F, Corredor C, Fletcher N, Tritapepe L, Lorini FL, Arcadipane A, Vieillard-Baron A, Cecconi M, Buggey J, Hoit BD, et al. Left ventricular systolic function evaluated by strain echocardiography and relationship with mortality in patients with severe sepsis or septic shock: A systematic review and Meta-Analysis. Crit Care. 2018;22:183. 10.1186/s13054-018-2113-y. 30075792 10.1186/s13054-018-2113-y PMC6091069

[38] Pruszczyk A, Zawadka M, Andruszkiewicz P, LaVia L, Herpain A, Sato R, Dugar S, Chew MS, Sanfilippo F. Mortality in patients with septic cardiomyopathy identified by longitudinal strain by speckle tracking echocardiography: an updated systematic review and Meta-Analysis with trial sequential analysis. Anaesth Crit Care Pain Med. 2024;43. 10.1016/J.ACCPM.2023.101339. 10.1016/j.accpm.2023.101339 38128732

[39] De Geer L, Oscarsson A, Engvall J. Variability in echocardiographic measurements of left ventricular function in septic shock patients. Cardiovasc Ultrasound. 2015;13. 10.1186/s12947-015-0015-6. 10.1186/s12947-015-0015-6 PMC4399417 25880324

[40] Nagueh SF, Sanborn DY, Oh JK, Anderson B, Billick K, Derumeaux G, Klein A, Koulogiannis K, Mitchell C, Shah A, et al. Recommendations for the evaluation of left ventricular diastolic function by echocardiography and for heart failure with preserved ejection fraction diagnosis: an update from the American society of echocardiography. J Am Soc Echocardiogr. 2025;38:537–69. 10.1016/j.echo.2025.03.011. 40617625 10.1016/j.echo.2025.03.011

[41] Sanfilippo F, Messina A, Scolletta S, Bignami E, Morelli A, Cecconi M, Landoni G, Romagnoli S. The CHEOPS bundle for the management of left ventricular diastolic dysfunction in critically ill patients: an experts’ opinion. Anaesth Crit Care Pain Med. 2023;42:101283. 10.1016/j.accpm.2023.101283. 37516408 10.1016/j.accpm.2023.101283

[42] Pécora M, Pastorini P, Farolini R, Burghi G, Hurtado FJ. Left ventricular systolic longitudinal strain in mechanically ventilated patients in the intensive care unit: assessment of global and chamber reproducibility. Intensive Care Med Exp. 2025;13:62. 10.1186/s40635-025-00770-8. 40526176 10.1186/s40635-025-00770-8 PMC12173981

[43] McErlane J, Shelley B, McCall P. Feasibility of 2-Dimensional speckle tracking echocardiography strain analysis of the right ventricle with Trans-Thoracic echocardiography in intensive care: A literature review and Meta-Analysis. Echo Res Pract. 2023;10:11. 10.1186/s44156-023-00021-0. 37469001 10.1186/s44156-023-00021-0 PMC10357770

[44] Lang RM, Badano LP, Mor-Avi V, Afilalo J, Armstrong A, Ernande L, Flachskampf FA, Foster E, Goldstein SA, Kuznetsova T, et al. Recommendations for cardiac chamber quantification by echocardiography in adults: an update from the American society of echocardiography and the European association of cardiovascular imaging. Eur Heart J Cardiovasc Imaging. 2015. 10.1093/ehjci/jev014. 25712077 10.1093/ehjci/jev014

[45] Skaarup KG, Lassen MCH, Johansen ND, Olsen FJ, Lind JN, Jørgensen PG, Jensen G, Schnohr P, Prescott E, Søgaard P, et al. Age- and Sex-Based normal values of Layer-Specific longitudinal and circumferential strain by speckle tracking echocardiography: the Copenhagen City heart study. Eur Heart J Cardiovasc Imaging. 2022;23:629–40. 10.1093/ehjci/jeab032. 33624014 10.1093/ehjci/jeab032

[46] Nyberg J, Jakobsen EO, Østvik A, Holte E, Stølen S, Lovstakken L, Grenne B, Dalen H. Echocardiographic reference ranges of global longitudinal strain for all cardiac chambers using Guideline-Directed dedicated views. JACC Cardiovasc Imaging. 2023;16:1516–31. 10.1016/j.jcmg.2023.08.011. 37921718 10.1016/j.jcmg.2023.08.011

[47] Mukherjee M, Rudski LG, Addetia K, Afilalo J, D’Alto M, Freed BH, Friend LB, Gargani L, Grapsa J, Hassoun PM, et al. Guidelines for the echocardiographic assessment of the right heart in adults and special considerations in pulmonary hypertension: recommendations from the American society of echocardiography. J Am Soc Echocardiogr. 2025;38:141–86. 10.1016/j.echo.2025.01.006. 40044341 10.1016/j.echo.2025.01.006

