# Supplementary material for: Sepsis-induced myocardial dysfunction diagnosed with strain versus non-strain echocardiography parameters: incidence, evolution and association with prognosis
Source: Ann Intensive Care. 2025 Sep 25;15:141. doi: 10.1186/s13613-025-01561-w (PMC12463772; doi:10.1186/s13613-025-01561-w)
Supplement: Supplementary file 2 — Supplementary Material 2. [file 13613_2025_1561_MOESM2_ESM.docx]

**PRICES CHECKLIST**


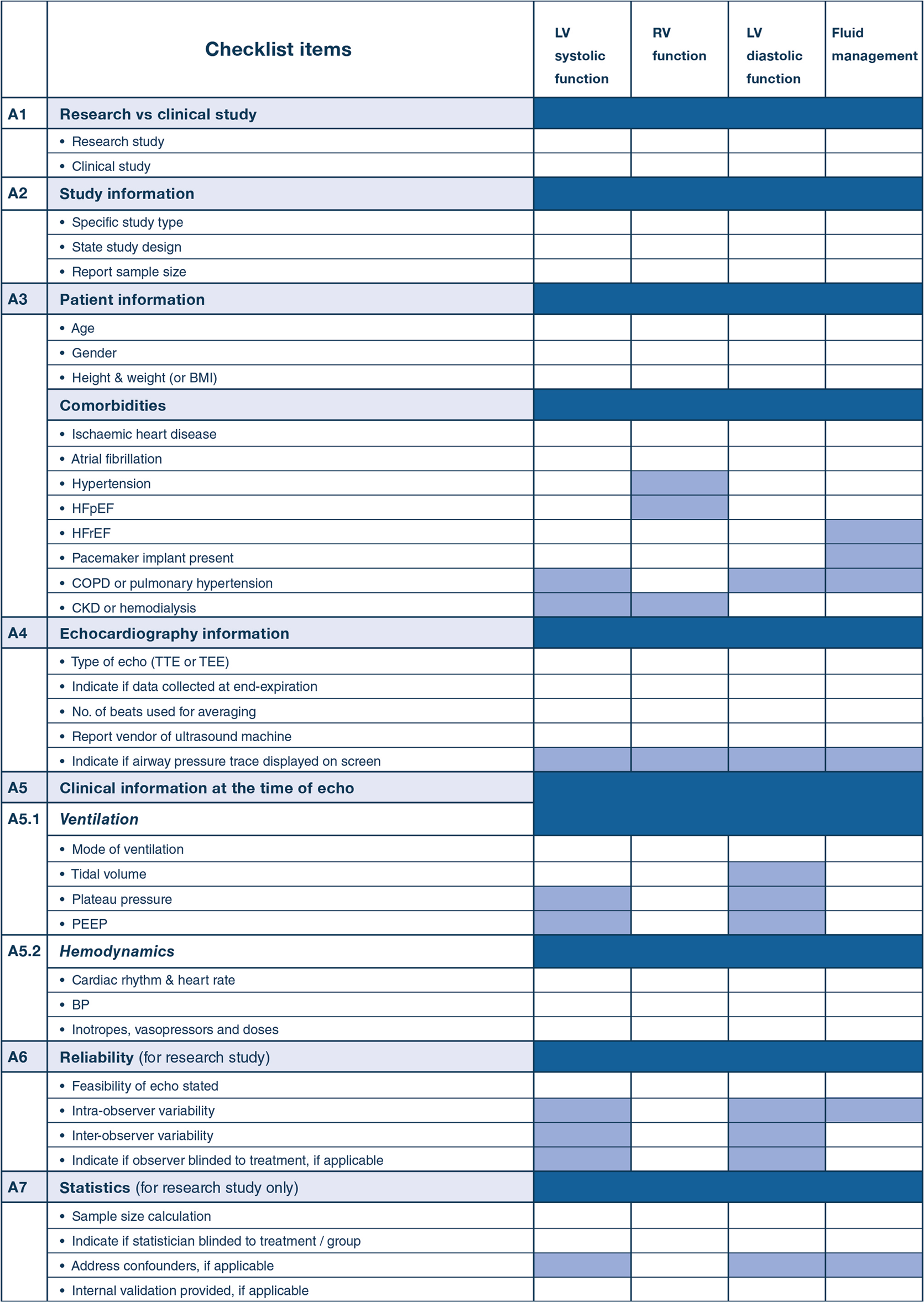


x x x

x x x

x x x

x x x

x x x

x x x

x x x

x x x

x x x

x x x

x x x

x x x

x x x

x x x

x x x

x x x

x x x

x x x

x x x

x x x

x x x

x x x

x x x

x x x

x x x

x x x

x x x

x x x

x x x

x x x

**
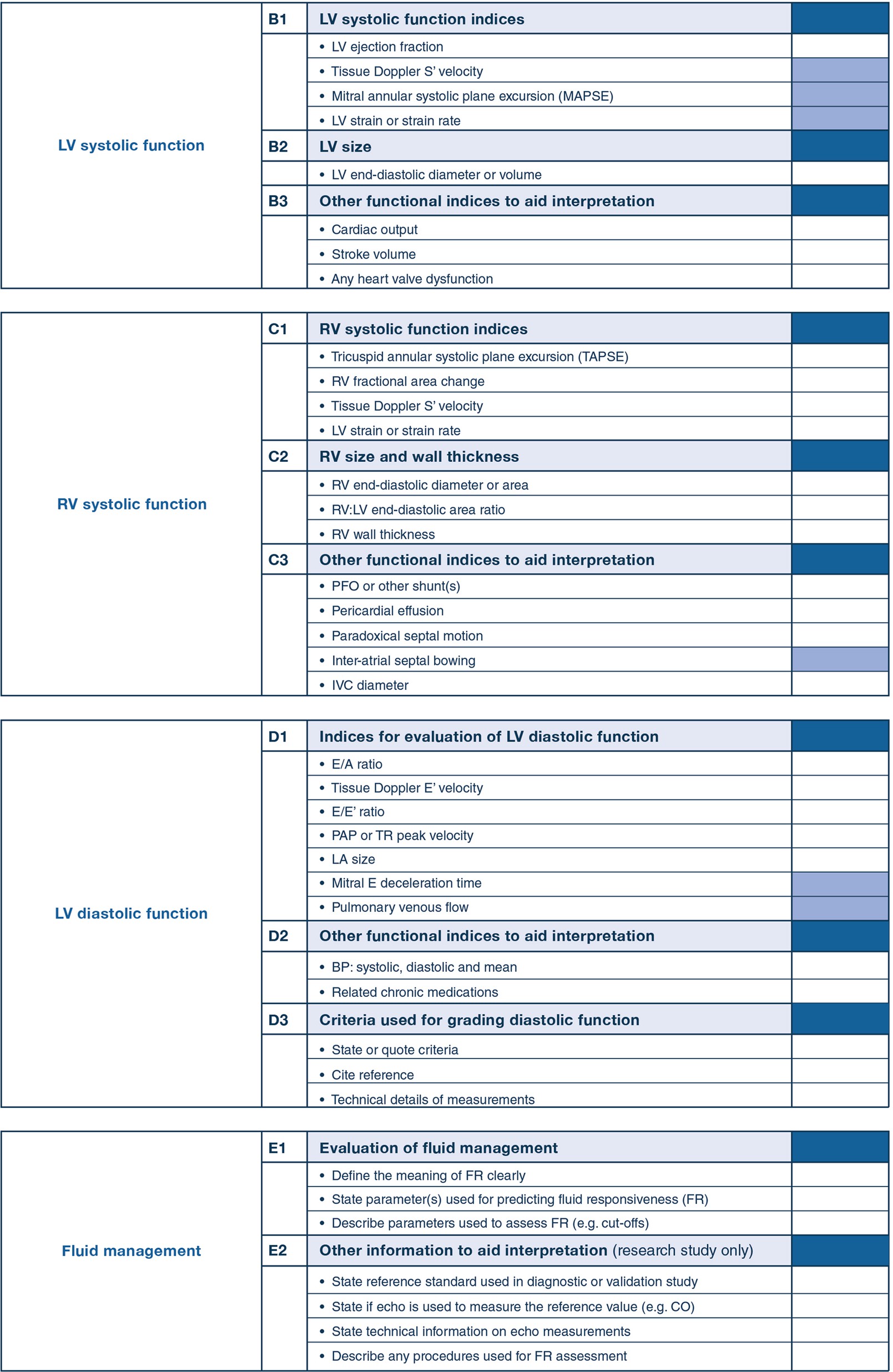
**

**x**

**x**

**x**

**x**

**x**

**x**

**x**

**x**

**x**

**x**

**x**

**x**

**x**

**x**

**x**

**x**

**x**

**x**

**x**

**x**

**x**

**x**

**x**
