# Supplementary material for: Sepsis-induced myocardial dysfunction diagnosed with strain versus non-strain echocardiography parameters: incidence, evolution and association with prognosis
Source: Ann Intensive Care. 2025 Sep 25;15:141. doi: 10.1186/s13613-025-01561-w (PMC12463772; doi:10.1186/s13613-025-01561-w)
Supplement: Supplementary file 3 — Supplementary Material 3. [file 13613_2025_1561_MOESM3_ESM.docx]

**Supplementary material**

**Table I. Cardiac biomarkers and echocardiographic evolution on the three timepoints – day 1, 7 and 30 - of septic shock patients.**

|  | Day 1 | Day 7 | Day 30 |
| --- | --- | --- | --- |
|  | n=98 | n=73 | n=54 |
| **Myocardial injury** |  |  |  |
| hsTn, ng/mL | 37 [17-158] | 13 [13-29] | 13 [13-27] |
| NT-proBNP, pg/mL | 3982 [824-12050] | 888 [308-2373] | 68 [45-472] |
| **Echocardiographic parameters** |  |  |  |
| **Left ventricle** |  |  |  |
| Dimensions |  |  |  |
| IVS, mm | 9 [8-10] | 10 [9-10] | 10 [9-10] |
| LVPW, mm | 9 [8-10] | 10 [9-10] | 10 [8-10] |
| LVEDV, mL | 98 [82-109] | 94 [79-109] | 89 [76-105] |
| LVESV, mL | 48 [37-60] | 45 [34-53] | 43 [37-50] |
| Function |  |  |  |
| LV S´, cm/s | 10 [8-13] | 11 [9-14] | 10 [8-12] |
| LVEF, % | 51 [41-58] | 56 [49-59] | 55 [48-59] |
| LV-GLS, % | 13 [11-18] | 16 [13-18] | 17 [14-18] |
| LVOT-VTI, cm | 19 [15-24] | 22 [19-25] | 22 [19-25] |
| **Left atrium** |  |  |  |
| E wave, cm/s | 79 [67-88] | 75 [63-88] | 71 [62-76] |
| A wave, cm/s | 56 [44-76] | 64 [51-81] | 62 [54-81] |
| Deceleration time, msec | 164 [122-217] | 172 [136-237] | 185 [164-266] |
| E/A | 1.3 [1-1.7] | 1 [0.8-1.4] | 1,1 [0.8-1.3] |
| e' lateral, cm/s | 10 [8-13] | 10 [9-14] | 10 [8-12] |
| e' septal, cm/s | 8 [7-10] | 8 [6-10] | 8 [7-10] |
| E/e' | 8,2 [6.3-9.8] | 7.1 [6-9.7] | 7,2 [6-8.6] |
| LAVi, mL/m2 | 25 [19-32] | 24 [17-33] | 22 [19-32] |
| PALS, % | 18 [12-24] | 20 [17-26] | 24 [17-27] |
| **Right ventricle** |  |  |  |
| RV-GLS, % | 16 [14-19] | 18 [16-22] | 19 [16-21] |
| TAPSE, mm | 19 [16-21] | 20 [18-22] | 21 [18-22] |
| FAC, % | 42 [36-48] | 45 [41-50] | 46 [41-50] |
| sPAP, mmHg | 36 [26-43] | 24 [16-32] | 20 [14-30] |

Data are presented as median [IQR] unless otherwise specified. IQR, interquartile range.

hsTn, high sensitivity troponin; NT-proBNP, N-terminal pro b-type natriuretic peptide; IVS, interventricular septum; LV, left ventricle; LVPW, LV posterior wall; LVEDD, LV end-diastolic diameter; LVESD, LV end-systolic diameter; LVEDV, LV end-diastolic volume; LVESV, LV end-systolic volume; LV S’, LV systolic myocardial velocity; LVEF, left ventricle ejection fraction; LV-GLS, LV global longitudinal strain; LVOT VTI, LV outflow tract velocity-time integral; E, peak early inflow velocity; A, peak late inflow velocity; E/A, peak early inflow velocity to peak late inflow velocity; e’, peak early longitudinal diastolic myocardial velocity; E/e’, peak early inflow velocity to peak early longitudinal diastolic myocardial velocity; LAVi, left atrial volume indexed; PALS, peak atrial longitudinal strain during the reservoir phase; RV, right ventricle; FAC, fractional area change; TAPSE, tricuspid annular plane systolic excursion; sPAP, systolic pulmonary artery pressure.

**Table II. Longitudinal evolution of cardiac function in patients admitted to intensive care unit with septic shock.**

| **Diagnosis** | **STE** | | | **Non-STE** | | |
| --- | --- | --- | --- | --- | --- | --- |
|  | **Day 1** | **Day 7** | **Day 30** | **Day 1** | **Day 7** | **Day 30** |
| **Normal** | 28 | 21 Normal  (D1 Normal=10;  D1 SIMD 11) | 21 Normal  (D1 Normal=9;  D1 SIMD=12) | 41 | 46 Normal  (D1 Normal=28;  D1 SIMD=18) | 36 Normal  (D1 Normal=23;  D1 SIMD=13) |
| **SIMD** | 70 | 52 SIMD  (D1 Normal=15;  D1 SIMD=37) | 33 SIMD  (D1 Normal=9;  D1 SIMD=24) | 57 | 27 SIMD  (D1 Normal=8;  D1 SIMD=19) | 18 SIMD  (D1 Normal=5;  D1 SIMD=13) |
| **Deaths** | - | 17 Deaths  (D1 Normal=2;  D1 SIMD=15) | 33 Deaths  (D1 Normal=5;  D1 SIMD=28) | - | 17 Deaths  (D1 Normal=3;  D1 SIMD=14) | 33 Deaths  (D1 Normal=6;  D1 SIMD=27) |
| **TOTAL** | **98** | **90** | **87** | **98** | **90** | **87** |
| **Missing** | 2 | 8 Missing  (D1 Normal=1;  D1 SIMD=7) | 11 Missing  (D1 Normal=5;  D1 SIMD=6) | 0 | 8 Missing  (D1 Normal=2;  D1 SIMD=6) | 11 Missing  (D1 Normal=7;  D1 SIMD=4) |

**Legend:** Diagnosis of normal cardiac function or septic-induced myocardial dysfunction (SIMD) is described according to both speckle-tracking echocardiography (STE) or conventional approach. Diagnosis is reported at three timepoints. For the cardiac function on day 7 and day 30, we report in brackets the diagnosis on day 1 (D1).
