# Supplementary material for: Sepsis-induced myocardial dysfunction diagnosed with strain versus non-strain echocardiography parameters: incidence, evolution and association with prognosis
Source: Ann Intensive Care. 2025 Sep 25;15:141. doi: 10.1186/s13613-025-01561-w (PMC12463772; doi:10.1186/s13613-025-01561-w)
Supplement: Supplementary file 4 — Supplementary Material 4. [file 13613_2025_1561_MOESM4_ESM.docx]

**Repeatability analysis**

- **Variables: LVEF_1_1, LVEF_1_2, LVEF_1_3**
- Single Measures ICC: .894 (95% CI: .215 - .969), Sig. < .001
- Average Measures ICC: .962 (95% CI: .450 - .989), Sig. < .001
- **Variables: LVEF_1_1, LVEF_2**
- Single Measures ICC: .833 (95% CI: -.015 - .961), Sig. < .001
- Average Measures ICC: .909 (95% CI: -.030 - .980), Sig. < .001
- **Variables: LV_GLS_1_1, LV_GLS_1_2, LV_GLS_1_3**
- Single Measures ICC: .916 (95% CI: .310 - .975), Sig. < .001
- Average Measures ICC: .970 (95% CI: .574 - .991), Sig. < .001
- **Variables: LV_GLS_1_1, LV_GLS_2**
- Single Measures ICC: .868 (95% CI: -.024 - .969), Sig. < .001
- Average Measures ICC: .929 (95% CI: -.049 - .984), Sig. < .001
- **Variables: GWE_1_1, GWE_1_2, GWE_1_3**
- Single Measures ICC: .672 (95% CI: .050 - .886), Sig. < .001
- Average Measures ICC: .860 (95% CI: .136 - .959), Sig. < .001
- **Variables: GWE_1_1, GWE_2**
- Single Measures ICC: .677 (95% CI: .154 - .915), Sig. < .001
- Average Measures ICC: .807 (95% CI: .162 - .956), Sig. < .001
- **Variables: EA_1_1, EA_1_2, EA_1_3**
- Single Measures ICC: .968 (95% CI: .640 - .990), Sig. < .001
- Average Measures ICC: .989 (95% CI: .842 - .997), Sig. < .001
- **Variables: EA_1_1, EA_2**
- Single Measures ICC: .946 (95% CI: .035 - .987), Sig. < .001
- Average Measures ICC: .972 (95% CI: .067 - .994), Sig. < .001
- **Variables: e_sep_1_1, e_sep_1_2, e_sep_1_3**
- Single Measures ICC: .965 (95% CI: .560 - .990), Sig. < .001
- Average Measures ICC: .988 (95% CI: .792 - .997), Sig. < .001
- **Variables: e_sep_1_1, e_sep_2**
- Single Measures ICC: .915 (95% CI: -.011 - .981), Sig. < .001
- Average Measures ICC: .956 (95% CI: -.023 - .990), Sig. < .001
- **Variables: Ee_1_1, Ee_1_2, Ee_1_3**
- Single Measures ICC: .986 (95% CI: .823 - .996), Sig. < .001
- Average Measures ICC: .995 (95% CI: .933 - .999), Sig. < .001
- **Variables: Ee_1_1, Ee_2**
- Single Measures ICC: .940 (95% CI: .041 - .986), Sig. < .001
- Average Measures ICC: .969 (95% CI: .078 - .993), Sig. < .001
- **Variables: PALS_1_1, PALS_1_2, PALS_1_3**
- Single Measures ICC: .962 (95% CI: .665 - .988), Sig. < .001
- Average Measures ICC: .987 (95% CI: .856 - .996), Sig. < .001
- **Variables: PALS_1_1, PALS_2**
- Single Measures ICC: .938 (95% CI: .070 - .985), Sig. < .001
- Average Measures ICC: .968 (95% CI: .130 - .992), Sig. < .001
- **Variables: RV_GLS_1_1, RV_GLS_1_2, RV_GLS_1_3**
- Single Measures ICC: .886 (95% CI: .216 - .966), Sig. < .001
- Average Measures ICC: .959 (95% CI: .452 - .988), Sig. < .001
- **Variables: RV_GLS_1_1, RV_GLS_2**
- Single Measures ICC: .839 (95% CI: -.020 - .962), Sig. < .001
- Average Measures ICC: .913 (95% CI: -.041 - .981), Sig. < .001
- **Variables: TAPSE_1_1, TAPSE_1_2, TAPSE_1_3**
- Single Measures ICC: .951 (95% CI: .391 - .986), Sig. < .001
- Average Measures ICC: .983 (95% CI: .659 - .995), Sig. < .001
- **Variables: TAPSE_1_1, TAPSE_2**
- Single Measures ICC: .849 (95% CI: -.013 - .965), Sig. < .001
- Average Measures ICC: .918 (95% CI: -.027 - .982), Sig. < .001
- **Variables: S_RV_1_1, S_RV_1_2, S_RV_1_3**
- Single Measures ICC: .966 (95% CI: .527 - .990), Sig. < .001
- Average Measures ICC: .988 (95% CI: .769 - .997), Sig. < .001
- **Variables: S_RV_1_1, S_RV_2**
- Single Measures ICC: .922 (95% CI: -.006 - .983), Sig. < .001
- Average Measures ICC: .960 (95% CI: -.013 - .991), Sig. < .001
- **Variables: PSAP_1_1, PSAP_1_2, PSAP_1_3**
- Single Measures ICC: .977 (95% CI: .705 - .993), Sig. < .001
- Average Measures ICC: .992 (95% CI: .878 - .998), Sig. < .001
- **Variables: PSAP_1_1, PSAP_2**
- Single Measures ICC: .933 (95% CI: .008 - .985), Sig. < .001
- Average Measures ICC: .966 (95% CI: .017 - .992), Sig. < .001
