# Supplementary material for: Sepsis-induced myocardial dysfunction diagnosed with strain versus non-strain echocardiography parameters: incidence, evolution and association with prognosis
Source: Ann Intensive Care. 2025 Sep 25;15:141. doi: 10.1186/s13613-025-01561-w (PMC12463772; doi:10.1186/s13613-025-01561-w)

**Univariable Logistic Regression of the 3 different cardiac dysfunction types: LVSD, LVDD and RVSD**

|  | | B | S.E. | Wald | Sig. | Exp(B) |
| --- | --- | --- | --- | --- | --- | --- |
| Step 0 | Constant | -,678 | ,214 | 10,058 | ,002 | ,508 |

|  | | | Score | Sig. |
| --- | --- | --- | --- | --- |
| Step 0 | Variables | LVSD (both) | 4,426 | ,035 |
|  |  | LVDD (both) | 4,337 | ,037 |
|  |  | RVSD (both) | ,006 | ,937 |
|  | Overall Statistics | | 7,696 | ,053 |

**Multivariable Logistic Regression including LVSD, LVDD and RVSD**

|  | | B | S.E. | Wald | Sig. | OR | 95% C.I.for OR | |
| --- | --- | --- | --- | --- | --- | --- | --- | --- |
|  |  |  |  |  |  |  | Lower | Upper |
| Step 1 | LVSD (both) | 1,120 | ,626 | 3,204 | ,073 | 3,064 | ,899 | 10,444 |
|  | LVDD (both) | ,506 | ,555 | ,832 | ,362 | 1,659 | ,559 | 4,920 |
|  | RVSD (both) | -,742 | ,534 | 1,930 | ,165 | ,476 | ,167 | 1,357 |
|  | Constant | -1,376 | ,425 | 10,500 | ,001 | ,253 |  |  |

| Model Summary | | | |
| --- | --- | --- | --- |
| Step | -2 Log likelihood | Cox & Snell R Square | Nagelkerke R Square |
| 1 | 118,120^a^ | ,070 | ,097 |
| a. Estimation terminated at iteration number 4 because parameter estimates changed by less than ,001. | | | |

| Hosmer and Lemeshow Test | | | |
| --- | --- | --- | --- |
| Step | Chi-square | df | Sig. |
| 1 | 7,388 | 4 | ,117 |

**Backward stepwise likelihood ratio Cox regression for STE and non-STE criteria of the several cardiac dysfunction types: LVSD, LVDD and RVSD:**

| Step | -2 Log Likelihood |  |  |
| --- | --- | --- | --- |
|  |  | Chi-square | Sig. |
| 1^a^ | 227,214 | 13,093 | ,042 |
| 2^b^ | 227,297 | 12,970 | ,024 |
| 3^c^ | 227,454 | 12,933 | ,012 |
| 4^d^ | 227,607 | 12,864 | ,005 |
| 5^e^ | 228,054 | 11,942 | ,003 |
| 6^f^ | 229,520 | 9,957 | ,002 |

|  | | **B** | **SE** | **Wald** | **Sig.** | **OR** | **95,0% CI for OR** | |
| --- | --- | --- | --- | --- | --- | --- | --- | --- |
|  |  |  |  |  |  |  | **Lower** | **Upper** |
| Step 1 | **LVSD-LVEF** | -0,430 | 0,637 | 0,456 | 0,500 | 0,650 | 0,187 | 2,267 |
|  | **LVSD-strain** | 0,202 | 0,686 | 0,087 | 0,768 | 1,224 | 0,319 | 4,694 |
|  | **LVDD-ASE/EACVI** | 1,306 | 0,705 | 3,432 | 0,064 | 3,692 | 0,927 | 14,704 |
|  | **LVDD-strain** | 0,133 | 0,461 | 0,083 | 0,774 | 1,142 | 0,463 | 2,818 |
|  | **RVSD-TAPSE** | 0,535 | 0,493 | 1,176 | 0,278 | 1,707 | 0,649 | 4,489 |
|  | **RVSD-strain** | -0,286 | 0,507 | 0,319 | 0,572 | 0,751 | 0,278 | 2,027 |
| Step 2 | **LVSD-LVEF** | -0,431 | 0,641 | 0,452 | 0,501 | 0,650 | 0,185 | 2,283 |
|  | **LVSD-strain** | 0,260 | 0,650 | 0,161 | 0,689 | 1,297 | 0,363 | 4,635 |
|  | **LVDD-ASE/EACVI** | 1,311 | ,700 | 3,513 | ,061 | 3,710 | ,942 | 14,616 |
|  | **RVSD-TAPSE** | 0,528 | 0,486 | 1,179 | 0,278 | 1,695 | 0,654 | 4,397 |
|  | **RVSD-strain** | -0,248 | 0,483 | 0,265 | 0,607 | 0,780 | 0,303 | 2,010 |
| Step 3 | **LVSD-LVEF** | -0,336 | 0,606 | 0,307 | 0,580 | 0,715 | 0,218 | 2,346 |
|  | **LVDD-ASE/EACVI** | 1,386 | 0,685 | 4,093 | 0,043 | 3,997 | 1,044 | 15,302 |
|  | **RVSD-TAPSE** | 0,512 | 0,494 | 1,071 | 0,301 | 1,668 | 0,633 | 4,394 |
|  | **RVSD-strain** | -0,178 | 0,459 | 0,150 | 0,698 | 0,837 | 0,340 | 2,058 |
| Step 4 | **LVSD-LVEF** | -0,402 | 0,580 | 0,480 | 0,489 | 0,669 | 0,215 | 2,086 |
|  | **LVDD-ASE/EACVI** | 1,413 | 0,682 | 4,286 | 0,038 | 4,108 | 1,078 | 15,649 |
|  | **RVSD-TAPSE** | 0,403 | 0,407 | 0,983 | 0,322 | 1,496 | 0,675 | 3,319 |
| Step 5 | **LVDD-ASE/EACVI** | 1,037 | 0,419 | 6,110 | 0,013 | 2,820 | 1,240 | 6,417 |
|  | **RVSD-TAPSE** | 0,476 | 0,387 | 1,513 | 0,219 | 1,610 | 0,754 | 3,437 |
| Step 6 | **LVDD-ASE/EACVI** | 1,186 | 0,396 | 8,956 | 0,003 | 3,273 | 1,506 | 7,116 |

**A Schoenfeld residuals analysis found no association between death and the partial residuals (p=0.078), with the following plots:**


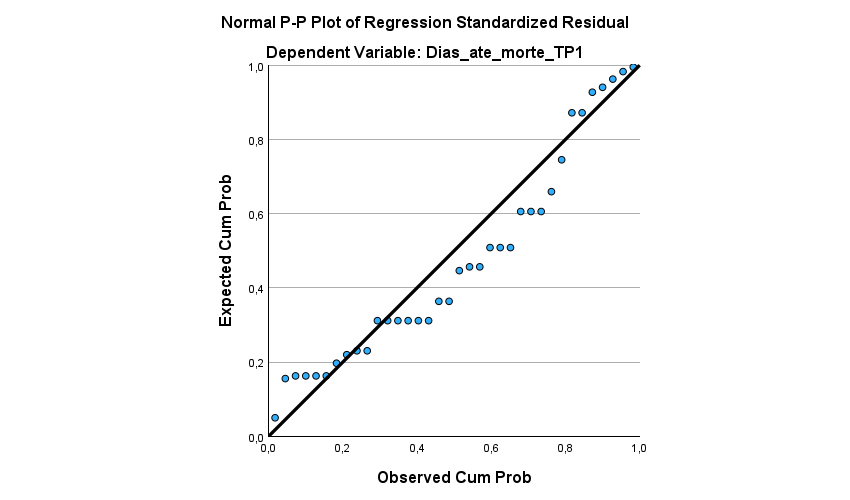


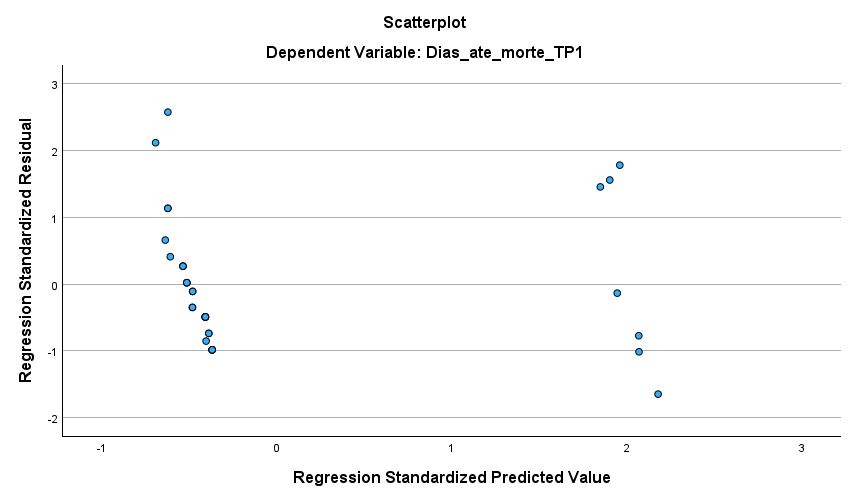

Supplement: Supplementary file 5 — Supplementary Material 5. [file 13613_2025_1561_MOESM5_ESM.docx]
